# Frozen Cord Blood Hematopoietic Stem Cells Differentiate into Higher Numbers of Functional Natural Killer Cells *In Vitro* than Mobilized Hematopoietic Stem Cells or Freshly Isolated Cord Blood Hematopoietic Stem Cells

**DOI:** 10.1371/journal.pone.0087086

**Published:** 2014-01-29

**Authors:** Martha Luevano, Anna Domogala, Michael Blundell, Nicola Jackson, Isabela Pedroza-Pacheco, Sophie Derniame, Michelle Escobedo-Cousin, Sergio Querol, Adrian Thrasher, Alejandro Madrigal, Aurore Saudemont

**Affiliations:** 1 University College London, Cancer Institute, London, United Kingdom; 2 Anthony Nolan Research Institute, London, United Kingdom; 3 Centre for Immunodeficiency, Molecular Immunology Unit, UCL Institute of Child Health, London, United Kingdom; 4 Programa Concordia Banc de Sang i Teixits, Barcelona, Spain; French Blood Institute, France

## Abstract

Adoptive natural killer (NK) cell therapy relies on the acquisition of large numbers of NK cells that are cytotoxic but not exhausted. NK cell differentiation from hematopoietic stem cells (HSC) has become an alluring option for NK cell therapy, with umbilical cord blood (UCB) and mobilized peripheral blood (PBCD34^+^) being the most accessible HSC sources as collection procedures are less invasive. In this study we compared the capacity of frozen or freshly isolated UCB hematopoietic stem cells (CBCD34^+^) and frozen PBCD34^+^ to generate NK cells *in vitro*. By modifying a previously published protocol, we showed that frozen CBCD34^+^ cultures generated higher NK cell numbers without loss of function compared to fresh CBCD34^+^ cultures. NK cells generated from CBCD34^+^ and PBCD34^+^ expressed low levels of killer-cell immunoglobulin-like receptors but high levels of activating receptors and of the myeloid marker CD33. However, blocking studies showed that CD33 expression did not impact on the functions of the generated cells. CBCD34^+^-NK cells exhibited increased capacity to secrete IFN-γ and kill K562 *in vitro* and *in vivo* as compared to PBCD34^+^-NK cells. Moreover, K562 killing by the generated NK cells could be further enhanced by IL-12 stimulation. Our data indicate that the use of frozen CBCD34^+^ for the production of NK cells *in vitro* results in higher cell numbers than PBCD34^+^, without jeopardizing their functionality, rendering them suitable for NK cell immunotherapy. The results presented here provide an optimal strategy to generate NK cells *in vitro* for immunotherapy that exhibit enhanced effector function when compared to alternate sources of HSC.

## Introduction

Natural Killer (NK) cells can kill infected or transformed cells without prior sensitization, making them an ideal cell product for immunotherapy [1]. NK cells can be directly isolated from umbilical cord blood (UCB) or peripheral blood (PB), or differentiated *in vitro* from hematopoietic stem cells (HSC). Several studies have explored the possibility of using NK cells for immunotherapy and highlighted the need to obtain high numbers of NK cells with optimal effector functions [2]–[4].

In this context, different sources of HSC have been used to generate NK cells *in vitro* including bone marrow (BM) [5], [6], human embryonic stem cells (hESC) [7], [8], mobilized peripheral blood stem cells (PBCD34^+^) [9], [10] and umbilical cord blood stem cells (CBCD34^+^) [11]–[14]. PBCD34^+^ and CBCD34^+^ are promising sources of HSC for this approach as PBCD34^+^ due to their accessibility. PBCD34+ have become more accessible due to the use of mobilizing agents and CBCD34^+^ have the advantage of non-invasive collection, less stringent human leucocyte antigen matching and off-the-shelf availability of more than 553,000 units from 47 UCB banks worldwide [15], [16]. The use of cryopreserved HSCs would present a convenient option for immunotherapy. However, different studies have reported that expansion of frozen HSC is often poor [17], with a decreased cell count and viability [18], while others have reported that frozen CBCD34^+^ can be used to generate NK cells [13] because of their high proliferative and clonogenic capacity [19]. In addition, with the rapid advancement in technology and new protocols supporting NK cell generation *in vitro*, the need to define which source of HSC, CBCD34^+^ or PBCD34^+^, fresh or frozen, is better for this approach is critical. Indeed, the possibility to generate high numbers of NK cells *in vitro* using CD34^+^ cells would facilitate multiple infusions and treatment of patients with large tumour burden, overcoming the limitations of low NK cell numbers and unfavourable activation state of blood-derived NK cells.

By modifying a published protocol [20], we showed that frozen CBCD34^+^ are a better HSC source to generate NK cells *in vitro* than fresh CBCD34^+^ and frozen PBCD34^+^, This approach generated higher numbers of NK cells with similar functional capacities, higher IFN-γ secretion and enhanced ability to kill K562 *in vitro* and *in vivo* without prior stimulation. To our knowledge, this is the first comprehensive study supporting the use of frozen CBCD34^+^ over fresh CBCD34^+^ and frozen PBCD34^+^ for NK cell generation *in vitro*.

## Results

### NK Cell Function and Phenotype are not Compromised by the Use of Frozen CBCD34^+^ or by the Removal of All Factors Except IL-15 at Week 3 of Culture

**Figure 1:**
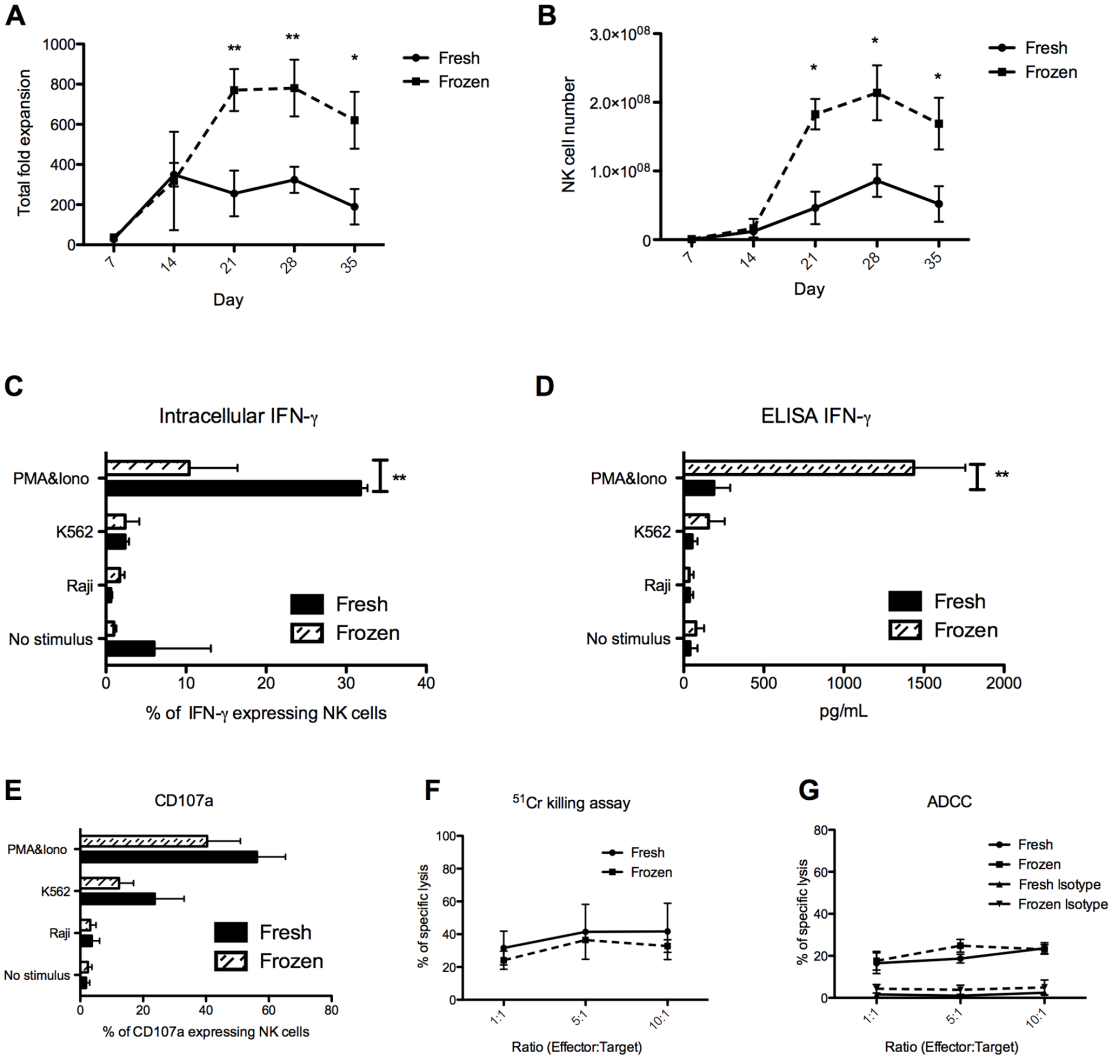
NK cell production from fresh and frozen CBCD34^+^. (**A**) Total fold expansion and (**B**) CD3^−^CD56^+^ NK cell number of fresh (n = 3) and frozen (n = 4) CBCD34^+^ cultures at different time points. (**C**) Intracellular expression of IFN-γ and (**D**) secreted IFN-γ as measured by ELISA for NK cells from fresh (n = 3) and frozen (n = 4) CBCD34^+^ cultures. (**E**) Degranulation assay using CD107a on CD56^+^CD3^−^ cells from fresh (n = 3) and frozen (n = 4) CBCD34^+^ cultures. NK cells from fresh (n = 3, solid line) and frozen (n = 4, dotted line) CBCD34^+^ cultures were co-incubated with ^51^Cr-labeled K562 cells (**F**) or ^51^Cr-labeled P815 cells (**G**) coated with anti-CD16 or an isotype control at different effector-target ratios in a standard 4 h ^51^Cr-release assay. The statistical analysis was performed using Mann-Whitney test, * *P*<0.05, ** *P<*0.005.

We first aimed to reduce the amount of cytokines used to produce NK cells *in vitro* based on a published protocol [20], using the same source of HSC, fresh CBCD34^+^, as described in this study. We analysed whether removing c-kit ligand (SCF), FLT-3 ligand and IL-7 and using only IL-15 from week 3 of culture would impact on the phenotype and functionality of the generated NK cells. No differences in fold expansion or NK cell number were found (Figure S1A–B). The phenotype of NK cells generated from cultures using all cytokines, compared to IL-15 only from week 3, was similar (Figure S1C). In addition, no differences were observed in NK cell degranulation after incubation of the generated cells with PMA&Iono or K562 cells (Figure S1D). Killing of K562 cells by NK cells generated under both conditions was similar (Figure S1E). Lastly, high levels of intracellular IFN-γ were detected in NK cells from both cultures (Figure S1F). The impact of using either fresh or frozen CBCD34^+^ on the repertoire and function of the NK cells generated was then assessed. Frozen CBCD34^+^ cultures showed higher expansion rates than fresh CBCD34^+^ cultures (*P<*0.05 for days 21, 28 and 35, figure 1A) and yielded higher NK cell numbers (figure 1B). Frozen CBCD34^+^-NK cells exhibited lower intracellular IFN-γ but higher secretion when stimulated with PMA&Ion (figure 1C–D). When studying their respective phenotypes, we only found significant differences in the expression of TRAIL, CXCR4 and granzyme B (Figure S2A–C). Nevertheless, we did not find any difference in degranulation, killing of K562 cells or ADCC by NK cells generated from fresh or frozen CBCD34+ (Figure 1E–G), suggesting that these phenotypic differences did not impact on the functions of the NK cells. Moreover, granzyme B messenger levels were comparable to those found in resting NK cells directly isolated from CB and PB (Figure S2D).

### CBCD34^+^ Cultures Generate Higher NK Cell Numbers than PBCD34^+^ Cultures

**Figure 2:**
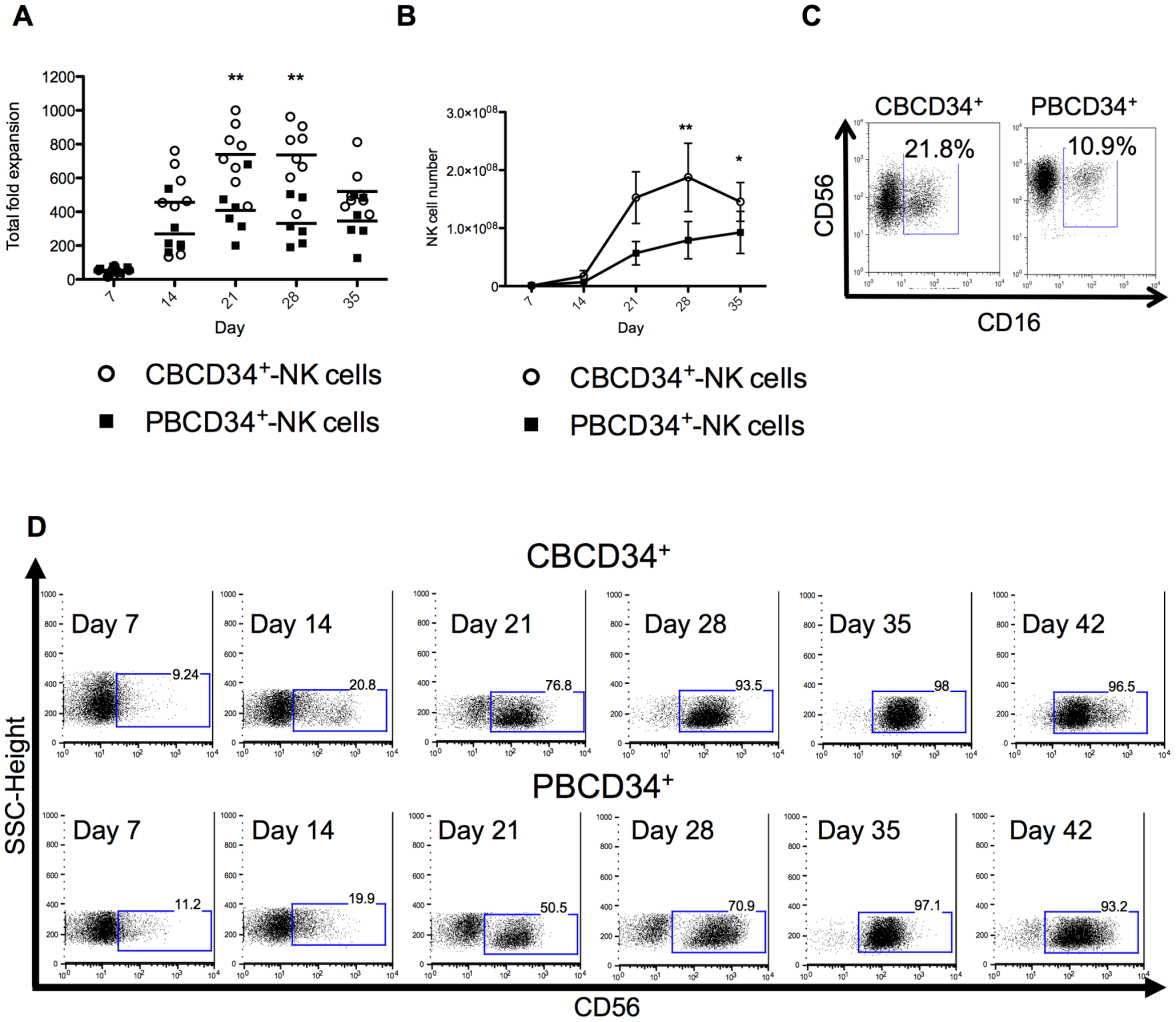
NK cell production from CBCD34^+^ and PBCD34^+^. (**A**) Total fold expansion and (**B**) CD3^−^CD56^+^ NK cell number of CBCD34^+^ (n = 8) and PBCD34^+^ (n = 6) cultures at different time points. (**C**) Representative plot of CD56 *vs* CD16 from the lymphocyte gate for CBCD34^+^ and PBCD34^+^ cultures. (**D**) Representative side scatter *vs* CD56^+^ plots for CBCD34^+^ (upper row) and PBCD34^+^ (bottom row) cultures at days 7, 14, 21, 28, 35 and 42. Mann-Whitney test was performed, * *P*<0.05, ** *P<*0.005.

CBCD34^+^ and PBCD34^+^ cells were cultured for up to 5 weeks using the modified protocol previously described and viability, cell number and NK cell yield were assessed on a weekly basis. CBCD34^+^ have been reported to have better proliferative capacity as compared to BM cells [21] and PBCD34^+^ cells [22]. Indeed, we observed a higher fold expansion in CBCD34^+^ cultures (day 21 and 28, *P*<0.05) in comparison to PBCD34^+^ cultures, but no significant difference between cultures was noted at day 35 (Figure 2A). In addition, we found higher NK cell numbers in CBCD34^+^ cultures than PBCD34^+^ cultures (*P*<0.05) (Figure 2B). NK cells from both cultures expressed CD16 (Figure 2C) but the acquisition of CD56 on the generated NK cells was slower for PBCD34^+^ cultures (Figure 2D). CD56 mean fluorescence intensity (MFI) had a tendency to be higher at day 28 in CBCD34^+^ cultures (Figure S3, p = 0.5287); nevertheless, CD56 expression was similar in both cultures by day 35. We did not detect T, B or NKT cells in either culture at any time point examined (data not shown), however a transitory CD14 expression was observed at day 14 (Figure S4).

### NK Cell Development is Delayed in PBCD34^+^ Cultures in Comparison to CBCD34^+^ Cultures

**Figure 3:**
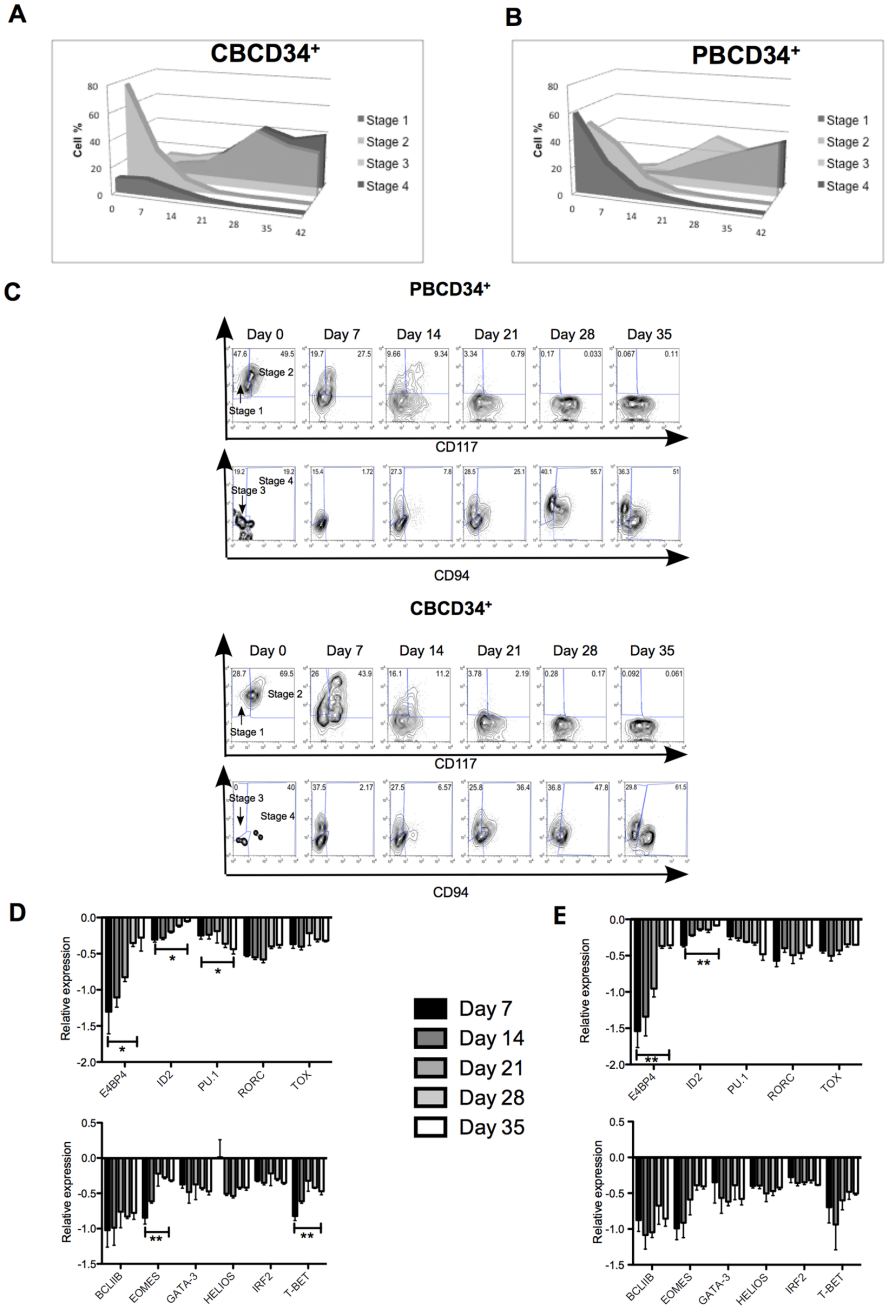
NK cell development in CBCD34^+^ and PBCD34^+^ cultures. NK cell stages 1–4 for one representative sample from CBCD34^+^ (n = 8) (**A**) and PBCD34^+^ (n = 6) (**B**) cultures. Percentages are gated from CD3^−^ cells according to the following NK cell stages: stage 1: CD34^+^CD117^−^CD94^−^, stage 2: CD34^+^CD117^+^CD94^−^, stage 3: CD34^−^CD117^+^CD94^−^ and stage 4: CD34^−^CD117^+/−^CD94^+^. (**C**) NK cell stages are shown for one representative sample for CBCD34^+^ (n = 8, upper panel) and PBCD34^+^ (n = 6, bottom panel) cultures at different time points. Stage 1 and 2 are from the CD3^−^CD94^−^ gate and stages 3 and 4 from the CD3^−^CD34^−^ gate. Transcriptional analysis for each time point is shown for transcription factors involved in NK cell differentiation (left panel) and maturation (right panel) for CBCD34^+^ (n = 4) (**D**) and PBCD34^+^ (n = 3) (**E**) cultures. Values are normalized using three reference genes. Higher ratio values correspond to less mRNA expression.

Although new evidence suggests that NK cells could differentiate from myeloid precursors [23], NK cells have traditionally been thought to originate from the lymphoid lineage. Haddad *et al*. have shown that the expression of CD45RA, CD34 and CD7 commits cells to the T/NK cell lineage [24]. Our study revealed that frozen CBCD34^+^ contained a higher percentage of CD45^+^CD7^+^ cells as compared to fresh CBCD34^+^ and PBCD34^+^ cells (Figure S5), suggesting that a higher number of NK cell committed progenitor cells were found in frozen CBCD34^+^. Additional analysis was performed based on the NK cell development model described by Freud and Caligiuri [25] whereby the surface expression of CD117, CD94 and CD34 was assessed in both CBCD34^+^ and PBCD34^+^ cultures. CBCD34^+^ expressed a high level of CD117 [26], therefore contain high numbers of stage 2 (CD34^+^CD117^+^CD94^−^) NK cell progenitors at day 0 (Figure 3A). In contrast, both stage 1 (CD34^+^CD117^−^CD94^−^) and 2 NK cell progenitors were found in PBCD34^+^ cultures at day 0 (Figure 3B). We observed a higher numbers of stage 3 NK cell progenitors (CD34^−^CD117^+^CD94^−^) in CBCD34^+^ cultures by week 2 compared to PBCD34^+^ cultures, probably due to the high initial levels of stage 2 progenitors in these cultures (Figure 3C). Unlike CBCD34^+^, NK cell development was slower in PBCD34^+^ cultures however the final numbers of stages 3 and 4 (CD34^−^CD117^−/+^CD94^+^) NK cells were similar at the end of the culture (Figure 3C).

NK cell development is orchestrated by molecular events that regulate the transition from one stage to another [27], [28]. Transcription factors (TFs) are key for this molecular regulation [29], [30]. We explored the expression of some TFs involved in NK cell differentiation (E4BP4, ID2, PU.1, RORC and TOX) and maturation (BCL11B, EOMES, GATA-3, HELIOS, IRF2 and T-BET) in the generated NK cells. In both cultures, PU.1 was more highly expressed during the first 3 weeks of culture with a subsequent decrease in expression (Figures 3D–E, day 7 versus day 35, p<0.05 in CBCD34^+^ and p = 0.0700 for PBCD34^+^). A tendency towards higher expression of ID2, RORC, TOX and BCL11B towards the end of the cultures was observed. E4BP4, EOMES and T-BET were more expressed at the end of the culture (day 7 versus day 35, p<0.05 in CBCD34^+^ and p = 0.0574 for PBCD34^+^). We observed a constant expression of IRF-2 and a variable expression GATA-3 during the whole culture. Interestingly, HELIOS expression was remarkably high in cells from CBCD34^+^ cultures at week 1, but not in cells from PBCD34^+^ cultures (Figure 3D).

### CBCD34^+^ Give Rise to CD56 Cells with a Distinctive Phenotype Characterized by Low DNAM-1 and Fas-L Expression but High TRAIL Expression

**Figure 4:**
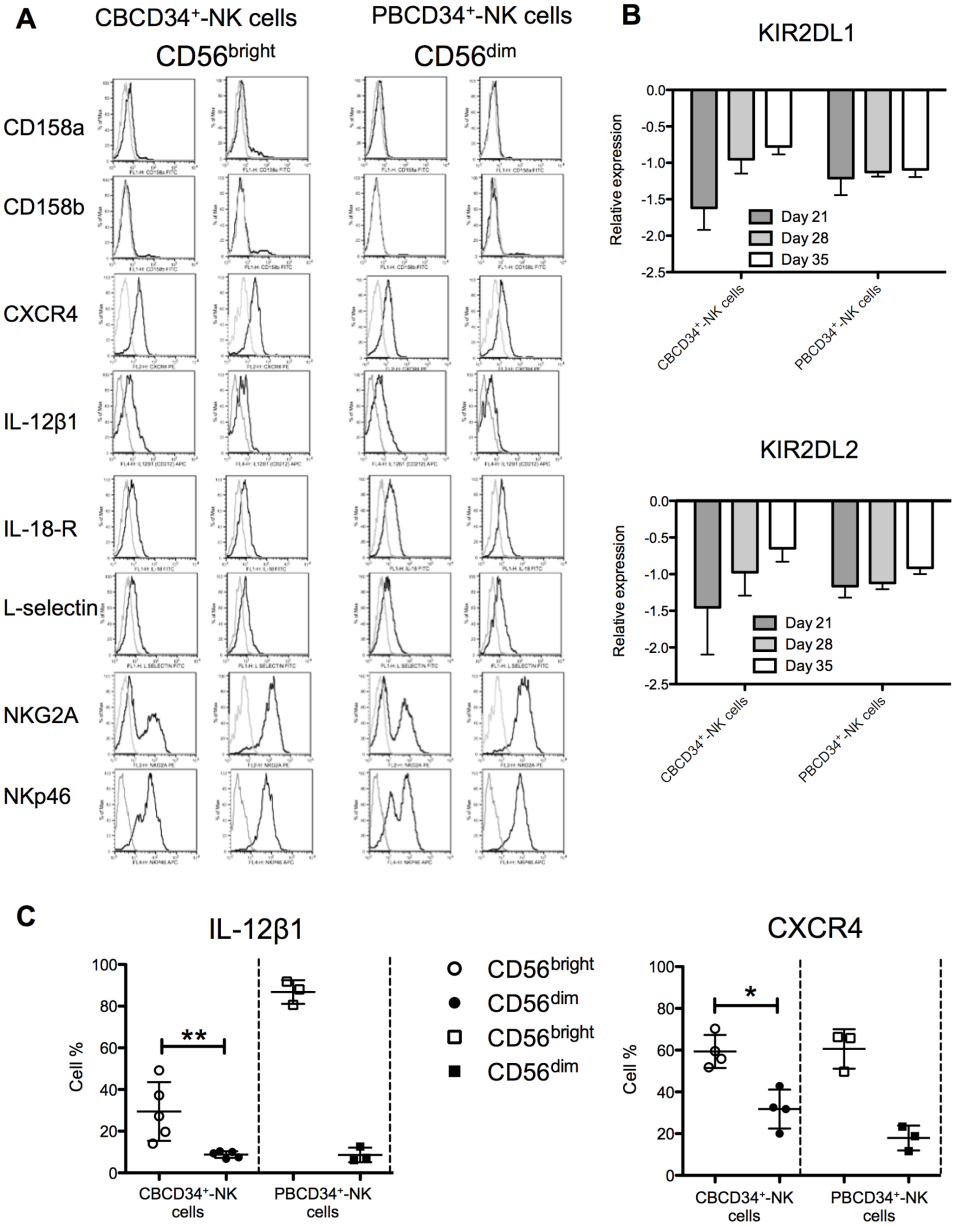
Phenotype of NK cells differentiated from CBCD34^+^ and PBCD34^+^ cultures. (**A**) Surface antigens were detected by flow cytometry. A representative sample for the expression of each antigen in the CD56^bright^ and CD56^dim^ subsets for CBCD34^+^ and PBCD34^+^ cultures is shown. (**B**) Transcriptional analysis for KIR2DL1 and KIR2DL2 at days 21, 28 and 35 is shown for NK cells from CBCD34^+^ (n = 4) and PBCD34^+^ (n = 3) cultures. Values are normalized using three reference genes. (**C**) Graphs depict the surface expression at day 35 of IL-12β1 and CXCR4 on NK cell subsets from frozen CBCD34^+^ (n = 4–5) and PBCD34^+^ (n = 3) cultures. Mann-Whitney test was performed, * *P*<0.05, ** *P*<0.005.

We further characterized the phenotype of the generated NK cells by analyzing the expression of different activating and inhibitory receptors (Figure S6A–D), interleukin receptors (Figure S6E), adhesion molecules (Figure S6F) and chemokine receptors (Figure S6G) by CD56^+^ cells in both cultures. Overall, NK cells from both cultures expressed the activating markers NKp30, NKp44, NKp46, CD48 and NKG2D, but lacked killer-cell immunoglobulin-like receptor (KIR) expression. The generated NK cells from both cultures also expressed IL-2α, IL-2β1, IL-12β1 receptors and NKG2A, as well as integrin β7, LFA-1 and high levels of CD49d. We observed a consistent CXCR4 expression but absence of CCR7 and CXCR7 expression on the generated NK cells from both cultures. CBCD34^+^ but not PBCD34^+^ cultures gave rise to CD56 cells with low expression of DNAM-1, Fas-L and IL-18-R, and high TRAIL expression (Figure S7). Expression of CD158a (KIR2DL1), CD158b (KIR2DL2/DL3), IL-18-R, L-selectin, NKG2A and NKp46 was higher in the CD56^dim^ subset (Figure 4A, *P*<0.05) for CBCD34^+^ cultures. Despite the low CD158a and CD158b expression observed by flow cytometry in the generated NK cells, messenger levels were increased from day 21 to 28 for CBCD34^+^-NK cells, whereas PBCD34^+^-NK cells maintained a constant level of messenger expression (Figure 4B). CD56^bright^ cells from CBCD34^+^ cultures expressed more CXCR4 and IL-12β1 (Figure 4C, *P*<0.05).

### CBCD34^+^-NK Cells Secrete More IFN-γ and have Better Killing Capacity than PBCD34^+^-NK Cells

**Figure 5:**
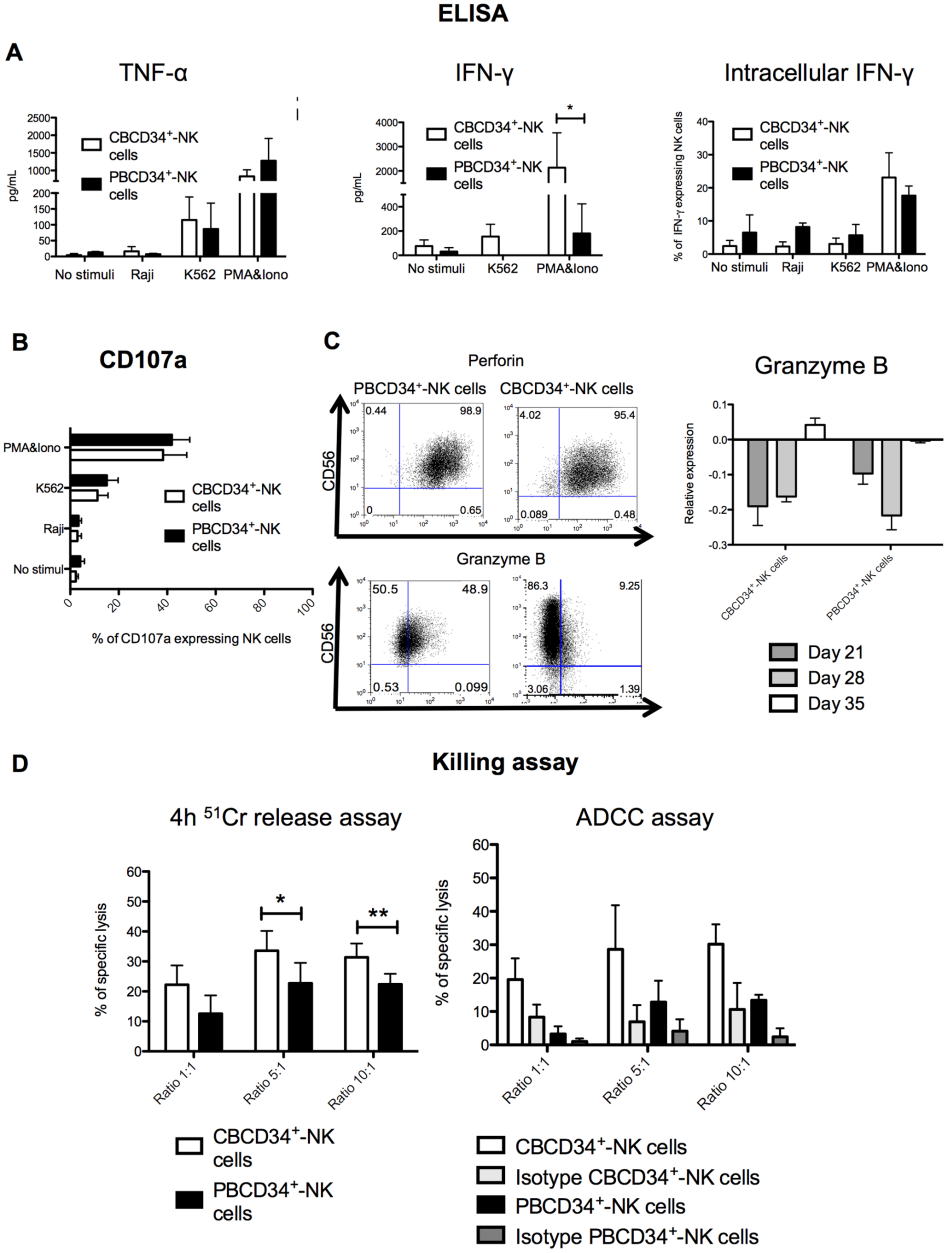
Killing capacity and cytokine production by NK cells from CBCD34^+^ and PBCD34^+^ cultures. (**A**) TNF-α and IFN-γ secretion by NK cells was measured by ELISA and intracellular expression of IFN-γ was detected by flow cytometry for CBCD34^+^ (n = 4) and PBCD34^+^ (n = 5) cultures. (**B**) CD107a degranulation assay using non-stimulated CD56^+^CD3^−^ cells or cells stimulated with K562, Raji or PMA&Iono for CBCD34^+^ (n = 9) and PBCD34^+^ (n = 6) cultures. (**C**) Representative intracellular staining for perforin and granzyme B at day 35 in NK cells from CBCD34^+^ (n = 9) and PBCD34^+^ (n = 6) cultures. The right panel shows granzyme B mRNA levels at days 21, 28 and 35 in NK cells from CBCD34^+^ (n = 5) and PBCD34^+^ (n = 3) cultures. (**D**) NK cells from CBCD34^+^ (n = 6) and PBCD34^+^ (n = 4) cultures were co-incubated with ^51^Cr-labeled K562 cells or ^51^Cr-labeled P815 cells coated with anti-CD16 or an isotype control at different effector-target ratios in a standard 4 h ^51^Cr-release assay. The statistical analysis was performed using Mann-Whitney test. * *P*<0.05, ** p<0.005.

A variety of assays were performed in order to assess the functions of the generated NK cells. First, we screened supernatants of the different cultures for the presence of cytokines and observed no statistical difference in TNF-α secretion between CBCD34^+^-NK cells and PBCD34^+^-NK cells irrespective of the stimuli used (Figure 5A). However, secretion of IFN-γ was significantly higher for NK cells from CBCD34^+^ cultures after stimulation with PMA&Iono (*P*<0.05). Interestingly, intracellular expression of IFN-γ did not differ between CBCD34^+^-NK cells and PBCD34^+^-NK cells (Figure 5A). The same number of NK cells were used for each IFN-γ assay suggesting that even though no difference in intracellular IFN-γ expression was found between CBCD34^+^-NK cells and PBCD34^+^-NK cells, CBCD34+ NK cells secreted higher levels of IFN-γ.

**Figure 6:**
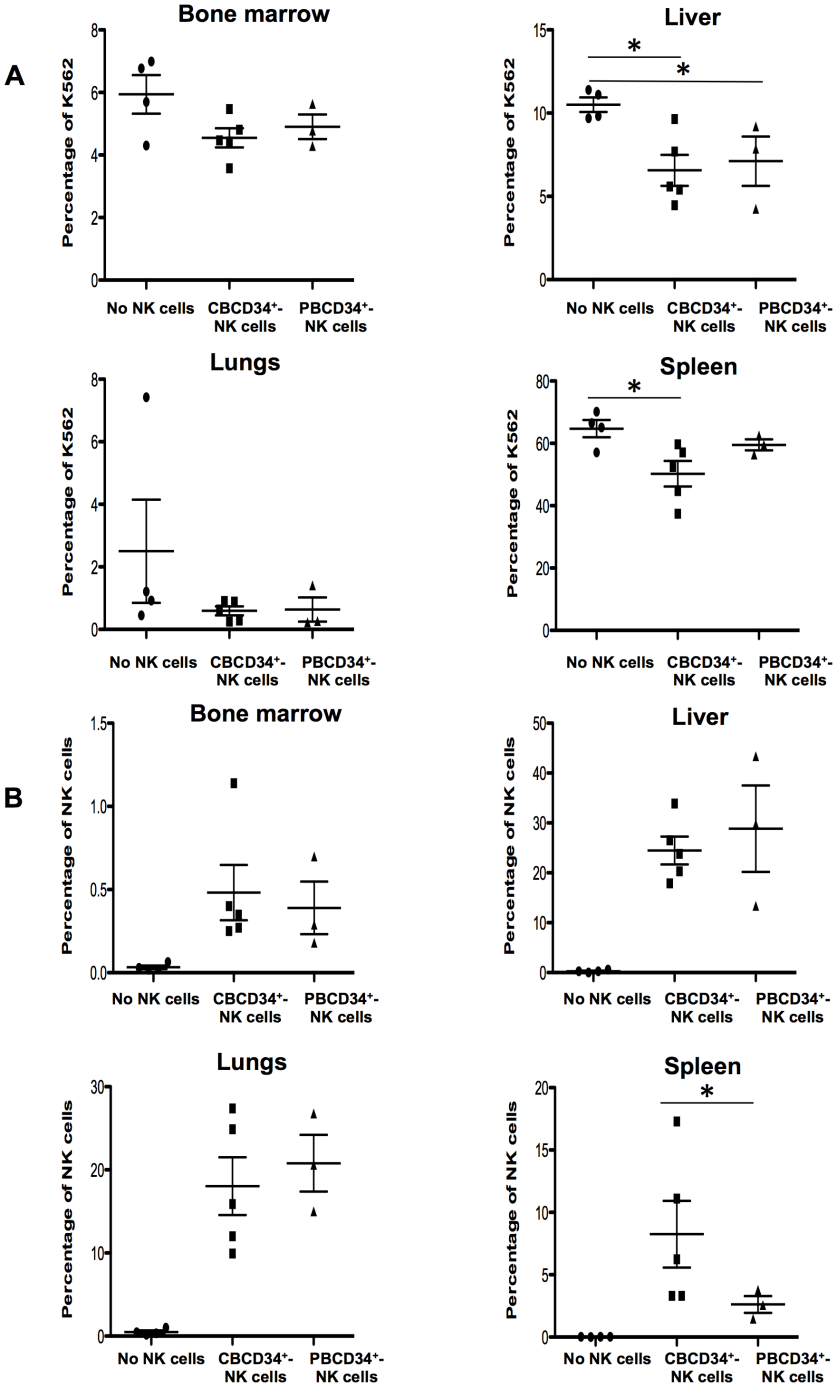
Killing of K562 *in vivo* by NK cells from CBCD34^+^ and PBCD34^+^ cultures. NSG mice were injected with GFP-K562 cells followed by CBCD34^+^-NK cells or PBCD34^+^-NK cells 24 h later. (**A**) Percentage of GFP-K562 cells detected in the BM, liver, lungs and spleen of NSG mice. (**B**) Percentage of NK cells detected in the BM, liver, lungs and spleen of NSG mice. The statistical analysis was performed using Mann-Whitney test. * *P*<0.05.

We detected a high percentage of degranulation by NK cells generated from both CBCD34^+^ and PBCD34^+^ when incubated with PMA&Iono or with K562 (Figure 5B). In addition, we found that perforin was highly expressed in CBCD34^+^ and PBCD34^+^ NK cells (Figure 5C), and that CBCD34^+^-NK cells exhibited low intracellular granzyme B expression. Nonetheless, granzyme B mRNA levels increased from day 21–35 in CBCD34^+^-NK cells, reaching similar values to those of PBCD34^+^-NK cells (Figure 5C) and were similar to those found in purified CB and PB NK cells (Figure S8). We then studied the killing capacity of the generated NK cells using a standard ^51^Cr release assay and ADCC. A higher killing capacity was observed for NK cells from CBCD34^+^ cultures for the effector:target ratios of 5∶1 and 10∶1 (*P*<0.05), as well as a tendency for better ADCC killing for CBCD34^+^-NK cells (Figure 5D, P = 0.0571). Finally, we assessed whether the generated NK cells could kill K562 *in vivo*. For this, NSG mice were injected with GFP-K562 cells followed by CBCD34^+^-NK cells or PBCD34^+^-NK cells 24 h later. As shown in Figure 6A, GFP-K562 cells could be detected 48 h after injection in the BM, liver, lungs and spleen of NSG mice. Reduced percentages of GFP-K562 cells were detected in the liver of NSG recipients injected with either type of NK cells as compared to control mice (*P*<0.05). The infusion of CBCD34^+^-NK cells, not of PBCD34^+^-NK cells, led to a significant decrease in the percentage of GFP-K562 cells found in the spleen as compared to NSG mice that did not receive NK cells (*P*<0.05), while no significant killing of GFP-K562 cells was observed in other tissues. In addition, CBCD34^+^-NK cells or PBCD34^+^-NK cells could be detected in different tissues of the injected mice (Figure 6B) with a notably higher percentage of NK cells found in the spleen of NSG mice injected with CBCD34^+^-NK cells in comparison to those animals injected with PBCD34^+^-NK cells (*P*<0.05).

### CD33 Expression does not Impact on the Function of the Generated NK Cells

**Figure 7:**
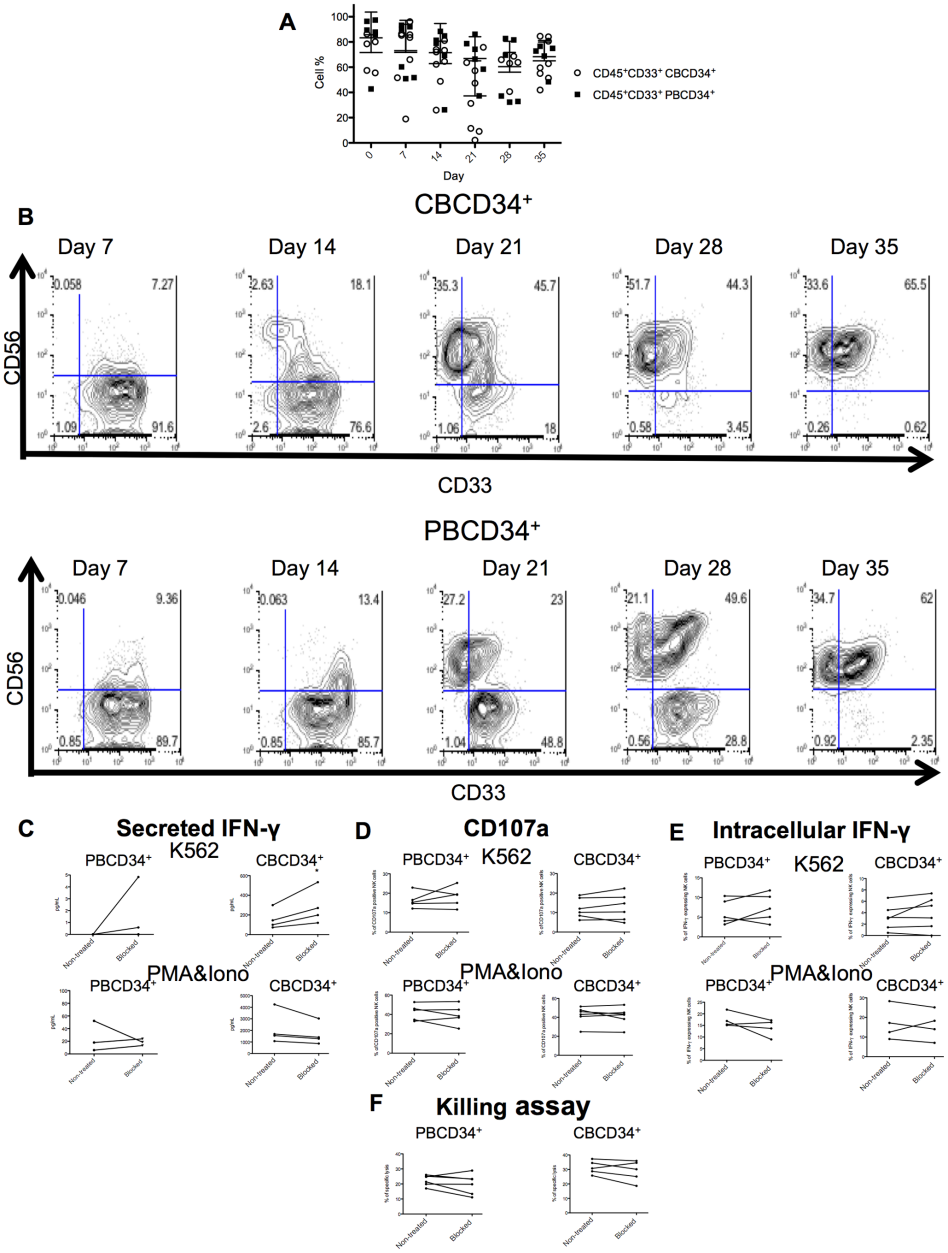
Expression of the myeloid marker CD33 and the effect of blocking CD33 on the differentiation of NK cells from CBCD34^+^ and PBCD34^+^. (**A**) Percentage of CD45^+^CD33^+^ cells at different time points for CBCD34^+^ (n = 8) and PBCD34^+^ (n = 6) cultures. (**B**) FACS plots showing CD33 expression against CD56 at different time points for CBCD34^+^ (n = 8) and PBCD34^+^ (n = 6) cultures. NK cells from CBCD34^+^ (n = 4–6) or PBCD34^+^ (n = 3–5) were incubated in the presence or not of a CD33 blocking antibody. Secreted IFN-γ (**C**), CD107a degranulation (**D**), intracellular IFN-γ (**E**) and NK cell killing *in vitro* of the malignant cell line K562 in a 10∶1 effector-target ratio (**F**) were evaluated. Statistical analysis was performed using paired T-test. * *P*<0.05.

Several works have highlighted the possibility of deriving NK cells from myeloid progenitors [23], [31], therefore we analyzed the expression of CD33, a myeloid marker, in both NK cell differentiation cultures. High levels of CD45^+^CD33^+^ cells were found in both cultures (Figure 7A). Furthermore, CD33^+^ cells acquired CD56 expression from day 7. In the case of CBCD34^+^ cells, CD33^+^ cells slowly became CD56^+^ overtime, contrary to PBCD34^+^, where separate populations could be distinguished at day 21 and 28 (Figure 7B). Nevertheless, at day 35 around 60% of CBCD34^+^ and PBCD34^+^ NK cells were CD56^+^CD33^+^. We then assessed whether blocking CD33 on the generated NK cells had an effect on function. IFN-γ secretion by CBCD34^+^-NK cells stimulated with K562 cells was significantly increased when CD33 was blocked (Figure 7C, *P*<0.05). However, we did not observe any significant difference in IFN-γ secretion after stimulation with PMA&Iono when CD33 was blocked on NK cells generated from either CBCD34^+^ or PBCD34^+^. In addition, no effect on expression of intracellular IFN-γ, CD107a expression or ^51^Cr release assay was observed (Figure 7C–F).

### CBCD34^+^-NK Cells Require Shorter Exposure to IL-12 than PBCD34^+^-NK Cells to Increase Immunoregulatory and Cytotoxic Functions

**Figure 8:**
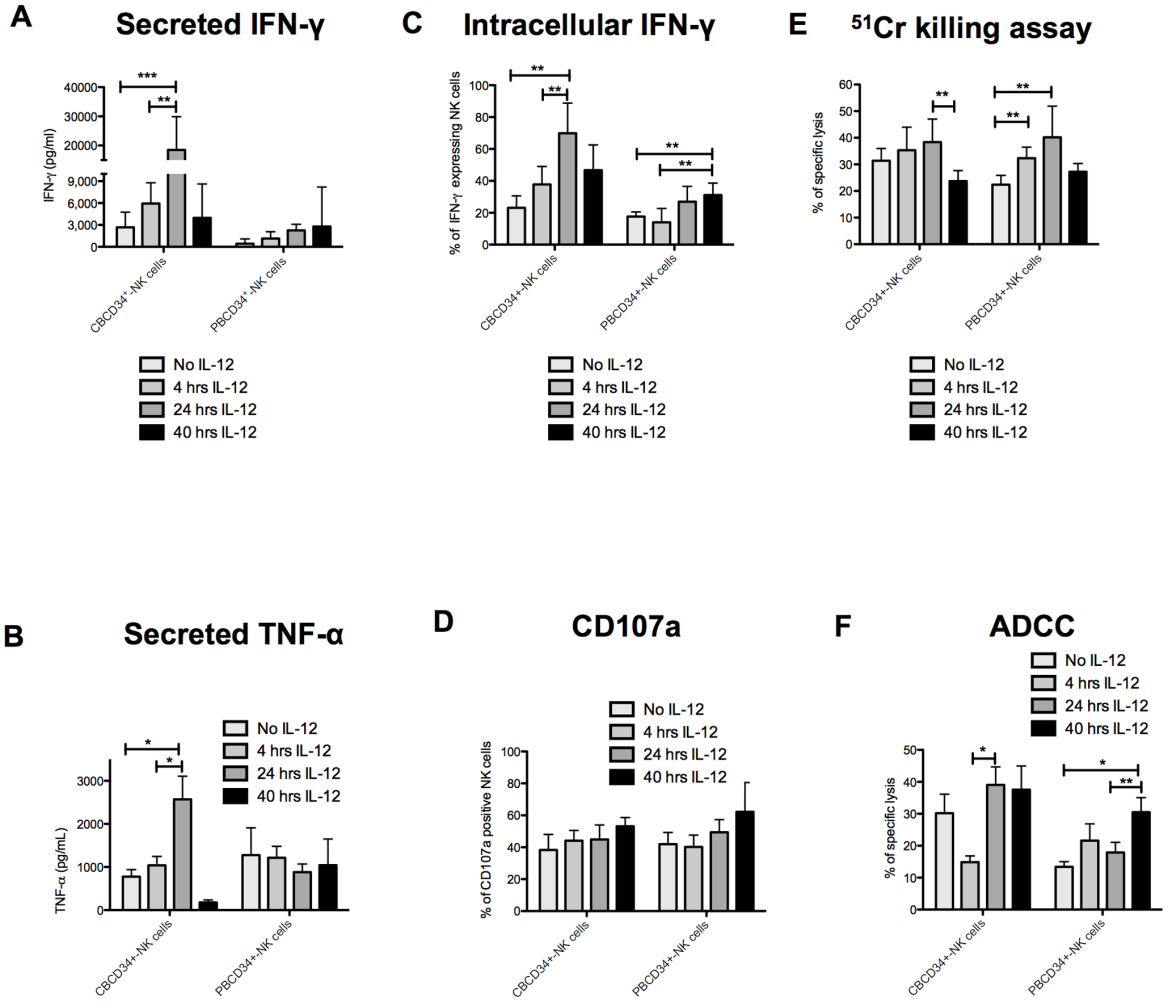
Effects of IL-12 stimulation on the function of the differentiated NK cells. NK cells were incubated with IL-12 for 4 h, 24 h or 40 h. (**A**) The figure illustrates the effect of IL-12 on the secretion of IFN-γ and (**B**) TNF-α measured by ELISA after incubation with PMA&Iono. (**C**) The graph depicts the intracellular expression of IFN-γ after incubation with PMA&Iono. (**D**) The graph shows CD107a degranulation after incubation with PMA&Iono. (**E**) NK cell killing capacity against ^51^Cr labeled K562 cells or (**F**) P815 cells coated with anti-CD16. The effector-target ratio used was 10∶1. Statistical analysis was performed using Mann-Whitney test. * *P*<0.05, ** p<0.005.

We decided to assess the effect of IL-12 on the generated NK cells because of its known ability to enhance NK cell cytotoxicity [32], secretion of IFN-γ [33] and TNF-α [32], and expression of adhesion molecules [34]. NK cells were harvested at day 35 from CBCD34^+^ and PBCD34^+^ cultures and incubated with IL-12 (20 ng/mL) for 4, 24 or 40 h. The use of IL-12 for 24 h in CBCD34^+^-NK cell cultures increased IFN-γ and TNF-α secretion as well as IFN-γ production (*P*<0.05, Figure 8A–C). Killing of K562 cells was also increased, although was not statistically significant after 4 h and 24 h incubation with IL-12. However, incubation with IL-12 for 40 h led to a decreased CBCD34^+^-NK cell killing capacity (*P*<0.05, Figure 8E). ADCC killing was reduced in CBCD34^+^-NK cells when incubated for 4 h with IL-12 which correlated with CD16 expression (Figure S9), but was increased when CBCD34^+^-NK cells were incubated for 24 h with IL-12 (*P*<0.05, Figure 7F). A subtle increase in secreted and intracellular IFN-γ in PBCD34^+^-NK cells (*P*<0.05, Figure 8A,C) was observed following incubation with IL-12, reaching its peak at 40 h. In the case of TNF-α, there was a slight decrease of secretion by NK cells after 24 and 40 h incubation with IL-12 (Figure 8B). PBCD34^+^-NK cells showed a better killing after 4 and 24 h IL-12 incubation (*P*<0.05) in ^51^Cr killing assay compared to 40 h IL-12 incubation (Figure 8E). CD107a expression increased slightly after IL-12 incubation for both cultures but was not significantly different (Figure 8D). Whilst the receptor repertoire of PBCD34^+^-NK cells did not change significantly after 40 h incubation with IL-12, expression of CD48, Fas-L and LFA-1 increased and expression of CD49d and NKG2D decreased on CBCD34^+^-NK cells (*P*<0.05, Figures S10 and S11).

## Discussion

NK cell immunotherapy relies on the use of high numbers of NK cells with optimal immunoregulatory and cytotoxic functions. In this report we first tested the impact of using a complete cytokine mix for the first three weeks of the culture followed by further maintenance with only IL-15 during the last three weeks of culture on NK cell production *in vitro*. The use of only IL-15 for the last weeks of culture would reduce costs and did not impact on the total fold expansion, NK cell numbers or on NK cell repertoire and functions. Using this modified protocol we then observed a higher and faster cell expansion from frozen CBCD34^+^ than from fresh CBCD34^+^ cultures. This observation differs from published works where expansion of frozen HSC was often poor [17] but agreed with another study showing that frozen UCB HSC could be used to generate NK cells [13]. Importantly, functional properties did not differ between NK cells generated from frozen and fresh CBCD34^+^ cells. The fact that the percentage of total committed NK cell progenitors (CD45^+^CD7^+^) was lower in fresh CBCD34^+^ and PBCD34^+^ cultures compared to frozen CBCD34^+^ cultures could explain the difference in fold expansion and NK cell numbers observed.

In terms of NK cell development, PBCD34^+^ initially contained a 1∶1 ratio of NK cell differentiation stages 1 and 2, whereas CBCD34^+^ contained mainly cells from stage 2. The impact of this different distribution at day 0 was reflected in the slower CD56 acquisition and delayed NK cell development in PBCD34^+^ cultures. When studying TF expression, we found that the only TF expressed highly during the first weeks of culture was PU.1, which regulates stem cell factor and IL-7 receptor expression, thus playing a key role in the transition of NK cell precursors to immature (iNK) and mature NK (mNK) cells [35]. Our data suggest that the importance of this factor is minimal at later stages of NK cell maturation, as previously described [30]. The low expression of BCL11B in our cultures could enable the expression of other TFs such E4BP4 and ID2 [36] that could restrict T-cell development. In line with previous reports that show that ID2 [37], E4BP4 [38] and TOX [39] are essential for the transition of iNK to mNK, these TFs were highly expressed during the last weeks of culture when this transition occurs. The maintenance of the mature NK cell pool in peripheral blood is in part regulated by the expression of IRF-2, a deficiency which causes accelerated apoptosis [40]. Although our system does not distinguish between biological compartments, we observed a constant and low expression of IRF-2 in the generated NK cells, suggesting that this factor is not involved directly in NK cell development, but could be important for NK cell survival throughout the culture. In addition, EOMES and T-BET, recently described as crucial TFs during the final steps of NK cell maturation [41], [42], were highly expressed during the last weeks of CBCD34^+^ and PBCD34^+^ cultures. This expression follows NK cell maturation in our system where acquisition of cytotoxic properties occurred.

Although CBCD34^+^ cultures acquired surface expression of the CD56 receptor faster than PBCD34^+^ cultures, CD56 MFI did not differ between generated NK cells from either culture. We found that CBCD34^+^-NK cells and PBCD34^+^-NK cells generated *in vitro* expressed a variety of activating markers, but did not express KIRs and the maturation marker CD57, suggesting an immature phenotype. In addition, we observed a lower expression of DNAM-1 and Fas-L, and higher expression of TRAIL in CBCD34^+^-NK cells as compared to PBCD34^+^-NK cells. The mechanisms responsible for these differences are unclear. We also found low KIR expression in both HSC cultures. Using a similar model, Dezell *et al*. suggest that early signals derived from the feeder layer from days 0–14 are needed for KIR acquisition [14]. It could be that additional signals are required in our system for KIR expression. In the same study the authors found higher CD16 expression on NK cells generated using a heparin-based system compared to those generated using the EL08.1D2 feeder layer [14]. The generation of CD56^+^CD16^+^ cells from either PBSC (100% CD16^+^) [43] or CBSC (24% CD16^+^) has been reported [44]. The discrepancy in CD16 expression between CBCD34^+^ and PBCD34^+^ cultures could be related to the maturation stage of the NK cells generated. It seems that the majority of CBCD34^+^-NK cells resemble CD56^dim^ NK cells (characterized by CD16 expression) as compared to CBCD34^+^-NK cells that exhibit more of a “CD56^bright^” phenotype.

Montaldo *et al*. also showed that CD161^−^CD56^−^LFA1^+^CD33^+^ cells could give rise to CD161^+^CD56^+^LFA1^−^NKp44^+^(CD33^−^) NK cells [45]. Interestingly, in our model, CD45^+^CD33^+^ cells also give rise to CD56^+^ cells. However, more than half of the generated NK cells co-expressed CD33 at day 35. Previous reports suggested an inhibitory role of CD33 on myeloid cells [46] and recently in NK cells derived from UCB (CD34^+^) [47] or from NK cell lines [48]. In our hands, blocking CD33 on NK cells only affected IFN-γ secretion by CBCD34^+^-NK cells when stimulated with K562 cells. We did not observe a significant impact on any of the other NK cell functions tested. However as some studies report rapid CD33 internalization after antibody binding [49], further studies are needed to definitively exclude a role for CD33 in regulating the functions of NK cells generated *in vitro*.

In line with other reports showing that *ex-vivo* generated NK cells from UCB are highly cytotoxic [11]–[13], CBCD34^+^-NK cells exhibited higher killing capacity and IFN-γ secretion compared to PBCD34^+^-NK cells, despite granzyme B expression being lower in CBCD34^+^-NK cells. Nevertheless, real time PCR analysis revealed high granzyme B messenger expression, similar to PBCD34^+^-NK cells, which is consistent with data showing that mRNA in NK cells can be rapidly translated when cellular activation occurs [50]. Incubation of generated NK cells with IL-12 increased granzyme B intracellular expression (data not shown), suggesting that additional activation signals are needed for granzyme B production by NK cells generated from frozen CBCD34^+^ cells. Importantly, the killing ability observed without further activation in the generated NK cells matches that observed for resting PB NK cells [51] and for a mixture of CD56^+^CD94^−^CD117^high^ and CD56^+^CD94^+^CD117^low/−^ populations analysed using a similar assay [20]. Furthermore, killing of K562 cells *in vivo* by both CBCD34^+^ and PBCD34^+^ NK cells was observed in the liver of NSG mice. However, only CBCD34^+^-NK cells and not PBCD34^+^-NK cells led to a significant killing of K562 cells in the spleen of NSG. Notably, this correlated with a higher percentage of NK cells in the spleen in recipients injected with CBCD34^+^-NK cells as compared to mice injected with PBCD34^+^ NK cells. This could be due to a better survival or proliferation of CBCD34^+^-NK cells, however this needs further investigation.

Interestingly, even though IL-12 receptor expression was similar for CBCD34^+^ and PBCD34^+^ NK cells, they did not respond similarly to this cytokine. CBCD34^+^-NK cells needed a shorter incubation with IL-12 in order to enhance their effector functions. Some studies have shown that UCB cells and in particular NK cells are more sensitive to IL-12 compared to BM or PB cells [52], [53]. IL-12 had a greater impact on the cytokine secretion of CBCD34^+^-NK cells compared to PBCD34^+^-NK cells. This is of great importance due to the key role that IFN-γ and TNF-α play in the protection against viral infections [54]. Nevertheless, IL-12 did not improve the killing capacity of CBCD34^+^-NK cells compared to PBCD34^+^-NK cells. The lower NKG2D and CD49d expression observed for CBCD34^+^-NK cells after 40 h of incubation with IL-12 could account for the lower killer capacity.

Importantly, we report here for the first time that higher NK cell numbers can be achieved using frozen CBCD34^+^ rather than fresh CBCD34^+^. This is of great relevance due to the non-invasive collection and off-the-shelf availability of UCB, offering a huge advantage over PBCD34^+^. Clinically, it is easier to utilize frozen cells, as they are often available off-the-shelf and can be preserved for long periods of time. If a thawed clinical grade UCB unit contains an average of 2.79×10^6^ cells [12], we could produce as many as 1.6×10^9^ NK cells. Alternatively, isolated HSC can be aliquoted and used to produce NK cells for multiple infusions at different time points.

In conclusion, we found that the use of frozen CBCD34^+^ to generate NK cells *in vitro* is a feasible option that offers the advantages of obtaining higher NK cell numbers with enhanced killing capacity and cytokine secretion, in addition to the possibility to further enhance NK cell functions with a short incubation with IL-12. To our knowledge, this is the most comprehensive study comparing these two HSC sources and supports the use of frozen CBCD34^+^ over fresh CBCD34^+^ for the generation of NK cells, highlighting the great potential for CBCD34^+^ for NK cell immunotherapy.

## Methods

### HSC Samples and Cell Lines

All UCB samples were obtained with prior consent and ethical committee approval from the Anthony Nolan Cord Blood bank (Research Ethics Committee reference 10/H0405/27). Fully informed written consent was obtained from pregnant mothers. The study had full ethical approval from the Anthony Nolan and Royal Free Hospital Research Ethics Committee. Frozen PBCD34^+^ samples were provided by Prof Kwee Yong, University College London Hospitals (UCLH) using chemotherapy/G-CSF. Informed written consent, with a protocol approved by the UCL/UCLH Committee on the Ethics of Human Research, was obtained. K562, GFP-K562 and Raji cells were cultured in RPMI containing 10% FBS.

### HSC Differentiation into NK Cells

UCB mononuclear cells were obtained by density centrifugation using Ficoll–Paque™ premium (GE Healthcare) and then HSC isolated using the CD34 MicroBead kit from Miltenyi Biotec [55]. CD34^+^ cells (500 per well) were plated in 96-well plates coated with irradiated EL08.1D2 cells and cultured as described by Grzywacz *et al*. [20] using either all growth factors for the whole culture duration, or for the first three weeks only followed by IL-15 alone at 50 ng/mL. Fresh medium and cytokines were added weekly after hemi-depletion. For IL-12 stimulation, 20 ng/mL IL-12 (Prospec) was added directly to each well at day 30 and incubated for 4, 24 or 40 h prior to analysis.

### Flow Cytometry

Cells were incubated at 4°C for 10 min or 45 min for labeling with anti-CXCR4 and anti-CXCR7 antibodies (R&D), washed and resuspended in 1X PBS containing 10% FBS. A FACSCalibur (Becton Dickinson) flow cytometer was used to acquire data and FlowJo software was used for data analysis. The following monoclonal antibodies were purchased from BD Biosciences: anti-CCR7, anti-CD3, anti-CD16, anti-CD33, anti-CD34, anti-CD45, anti-CD48, anti-CD56, anti-CD62L, anti-CD94, anti-CD95, anti-CD107a, anti-CD117, anti-CD158a, anti-CD158b, anti-CD226, anti-granzyme B, anti-IFN-γ, anti-NKp30, anti-NKp46, anti-TRAIL, anti-perforin; from Biolegend: anti-CD57, anti-NKp44 and anti-NKp80; 7AAD was purchased from Immunotools; anti-NKG2D from Miltenyi Biotec and anti-2B4, anti-CD49d, anti-integrin β7, anti-CXCR1, anti-CXCR7, anti-CCR5 and anti-CCR6 from R&D. Anti-NKG2A was purchased from Beckman Coulter. For intracellular staining, 2×10^5^ NK cells were incubated with RPMI, K562 or Raji cells (ratio 1∶1) or 100 ng/mL phorbol 12-myristate 13-acetate and 1 µg/mL Ionomycin (PMA&Iono) for 1 h at 37°C. GolgiStop™ was then added and incubated for 4 h. Thereafter, surface staining was performed for CD3, CD16 and CD56, followed by intracellular staining for IFN-γ or an isotype control using permeabilization/fixation buffer (BD cytofix/cytoperm plus). For granzyme B and perforin detection, NK cells were directly stained without prior stimulation.

### Degranulation and Cytotoxicity Assay

2×10^5^ NK cells were incubated for 2 h at 37°C with medium, K562 or Raji cells in a 1∶1 ratio or PMA&Iono. Cells were stained with anti-CD56, -CD3 and -CD16 and then with anti-CD107a or the appropriate isotype control for 45 min at 4°C. For killing assays, K562 cells were labeled for 45 min with 100 µCi ^51^Chromium (^51^Cr)/1×10^6^ cells at 37°C. After 4 h co-culture with NK cells, supernatants were collected to measure ^51^Cr release. For antibody dependent cell cytotoxicity (ADCC) assays, P815 cells were incubated for 30 min at 37°C with anti-CD16 antibody (1 µg/mL) or the respective isotype at the same concentration, prior to co-culture with effector cells. CD33 blocking was performed according to a previously published protocol [48].

### ELISA

The Human Interferon–gamma Ready-set-go!® and Human TNF-alpha Ready-SET-Go!® ELISA kits from eBioscience were used according to the manufacturer’s instructions.

### Quantitative PCR

RNA was extracted using the RNAeasy Minikit (Qiagen). cDNA was synthesized using random primers (Promega) and the SuperScript II First-Strand Synthesis system (Invitrogen). The primer sequences and concentrations used are shown in Table S1. Quantitative real-time PCR was performed using SYBR Green technology (Primer Design Ltd) and actin β (ACTB), topoisomerase (DNA) I (TOP1) and ubiquitin C (UBC) (Primer Design Ltd) were used as reference genes. All reactions were performed as follows: 2 min 50°C, 10 min 95°C, then 50 cycles of 15 sec 95°C and 1 min 60°C. The quantity of mRNA was normalized to that of the mean of the three reference genes.

### Animal Experiments

NOD/SCID IL-2Rγnull (NSG) mice were irradiated at 3.75 Gy and injected intravenously with 1×10^6^ GFP-K562 cells followed by 20×10^6^ CBCD34^+^-NK cells or PBCD34^+^-NK cells 24 h later. Control mice were injected with GFP-K562 cells only. All mice were culled 48 h post injection of GFP-K562 cells and then the presence of the GFP-K562 and NK cells was assessed in different tissues. All experiments were performed in agreement with Home Office regulations (project license 80/1293).

### Statistics

Statistical comparisons were performed with GraphPad Prism software (GraphPad Software) using the nonparametric Mann–Whitney test or paired t-test. Results are presented as means ± standard deviation (SD), *P*<0.05 were considered statistically significant.

## Supporting Information

Figure S1 **NK cell production from fresh CBCD34+ cultures using different cytokine cocktails.** (**A**) Total fold expansion and (**B**) cell number of CD3^−^CD56^+^ cells of fresh CBCD34^+^ cultures using all cytokines (n = 3, solid line) or only IL-15 (n = 3, dotted line). (**C**) Expression of NK cell markers by NK cells from fresh CBCD34^+^ cultures using all cytokines (n = 3) or only IL-15 (n = 3). (**D**) Degranulation assay using CD107a on NK cells from fresh CBCD34^+^ cultures using all cytokines (n = 3) or only IL-15 (n = 3). **(E)** NK cells from fresh CBCD34^+^ cultures using all cytokines (n = 3) or only IL-15 (n = 3) were co-incubated with ^51^Cr-labeled K562 cells at different effector-target ratios in a standard 4 h ^51^Cr-release assay. **(F)** Produciton of IFN-γ by NK cells from fresh CBCD34^+^ cultures using all cytokines (n = 3) or only IL-15 (n = 3).
(TIFF)

Figure S2 **Characterization of fresh and frozen CBCD34^+^-NK cells.** The graph shows expression of (**A**) NK cell markers, (**B**) intracellular granzyme B and perforin and (**C**) chemokine receptors by NK cells from fresh (n = 3) and frozen (n = 4) CBCD34^+^ cultures. (**D**) Transcriptional analysis of granzyme B mRNA in NK cells from different sources. Values were normalized using three reference genes. Higher ratio values correspond to less mRNA expression. Mann-Whitney test was performed. * *P*<0.05, ** *P*<0.005.
(TIFF)

Figure S3 **Expression of CD56 during HSC cultures.** Expression of CD56 as measured by MFI by NK cells from CBCD34^+^ (n = 9) and PBCD34^+^ (n = 6) cultures at different time points.
(TIFF)

Figure S4 **Expression of CD14 during HSC cultures.** A representative FACS plot (CD56 *vs* CD14) from CBCD34+ and PBCD34^+^ cultures at days 14 and 35 showing expression of the monocyte marker CD14.
(TIFF)

Figure S5 **Frequency of CD45^+^CD7^+^ cells during HSC cultures.** Percentages of CD45^+^CD7^+^ progenitor cells in fresh (n = 3) and frozen CBCD34^+^ (n = 9) and PBCD34^+^ (n = 6) cultures at different time points.
(TIFF)

Figure S6 **Phenotypic characterization of NK cells from CBCD34^+^ and PBCD34^+^ cultures.** NK cells from CBCD34^+^ (n = 9, open circles) and PBCD34^+^ (n = 6, black squares) cultures were harvested at day 35 and stained with antibodies against the indicated surface antigens. For each marker, the median and standard deviation is presented for (**A**) Natural cytotoxicity receptors (NCRs), (**B**) co-stimulatory molecules, (**C**) inhibitory markers, (**D**) activating markers, (**E**) interleukin receptors, (**F**) adhesion molecules and (**G**) chemokine receptors on CD56^+^CD3^−^ cells from both cultures. The statistical analysis was performed using Mann-Whitney test. * *P*<0.05, ** *P*<0.005.
(TIFF)

Figure S7 **Expression of TRAIL, DNAM-1, Fas-L and IL-18R by NK cells from CBCD34^+^ and PBCD34^+^ cultures.** Representative FACS plots of CD56 *vs* CD14, CD56 *vs* DNAM-1, CD56 *vs* Fas-L and CD56 *vs* IL-18R of NK cells from CBCD34^+^ and PBCD34^+^ cultures.
(TIFF)

Figure S8 **Granzyme B expression by NK cells from CBCD34^+^ and PBCD34^+^ cultures.** (**A**) Transcriptional analysis of granzyme B mRNA in NK cells from CB, PB, CBCD34^+^ cultures and PBCD34^+^ cultures. Values were normalized using three reference genes. Higher ratio values correspond to less mRNA expression. Representative FACS plots of CD56 *vs* Granzyme B (**B**), CD56 *vs* Perforin (**C**) or the corresponding isotype control of NK cells from CBCD34^+^ and PBCD34^+^ cultures.
(TIFF)

Figure S9 **Effect of IL-12 on CD16 expression by the differentiated NK cells.** The figure shows a representative example of CD56^+^CD3^−^ cells from (**A**) CBCD34^+^ and (**B**) PBCD34^+^ cultures prior to and after incubation with IL-12 for 4, 24 or 40 h. The plots show CD56 *vs* CD16 for each time point. Percentages shown represent CD56^+^CD16^+^ cells.
(TIFF)

Figure S10 **Effect of IL-12 on the expression of activating and inhibitory receptors by differentiated NK cells.** NK cells from (**A**) CBCD34^+^ (n = 9) and (**B**) PBCD34^+^ (n = 6) cultures were incubated with IL-12 for 40 h. After incubation, cells were collected and labelled with antibodies against the indicated surface antigens. Statistical analysis was performed using Mann-Whitney test. * *P*<0.05.
(TIFF)

Figure S11 **Effect of IL-12 on the expression of chemokine receptors and adhesion molecules by the differentiated NK cells.** NK cells from (**A**) CBCD34^+^ (n = 9) and (**B**) PBCD34^+^ (n = 6) cultures were incubated with IL-12 for 40 h. After incubation, cells were collected and labelled with antibodies against the indicated surface antigens or an isotype control. Statistical analysis was performed using Mann-Whitney test. * *P*<0.05.
(TIFF)

Table S1 **Primer Sequences.**
(TIFF)
