# Supplementary figures and images for: Frozen Cord Blood Hematopoietic Stem Cells Differentiate into Higher Numbers of Functional Natural Killer Cells In Vitro than Mobilized Hematopoietic Stem Cells or Freshly Isolated Cord Blood Hematopoietic Stem Cells
Source: PLoS One. 2014 Jan 29;9(1):e87086. doi: 10.1371/journal.pone.0087086 (PMC3906137; doi:10.1371/journal.pone.0087086)

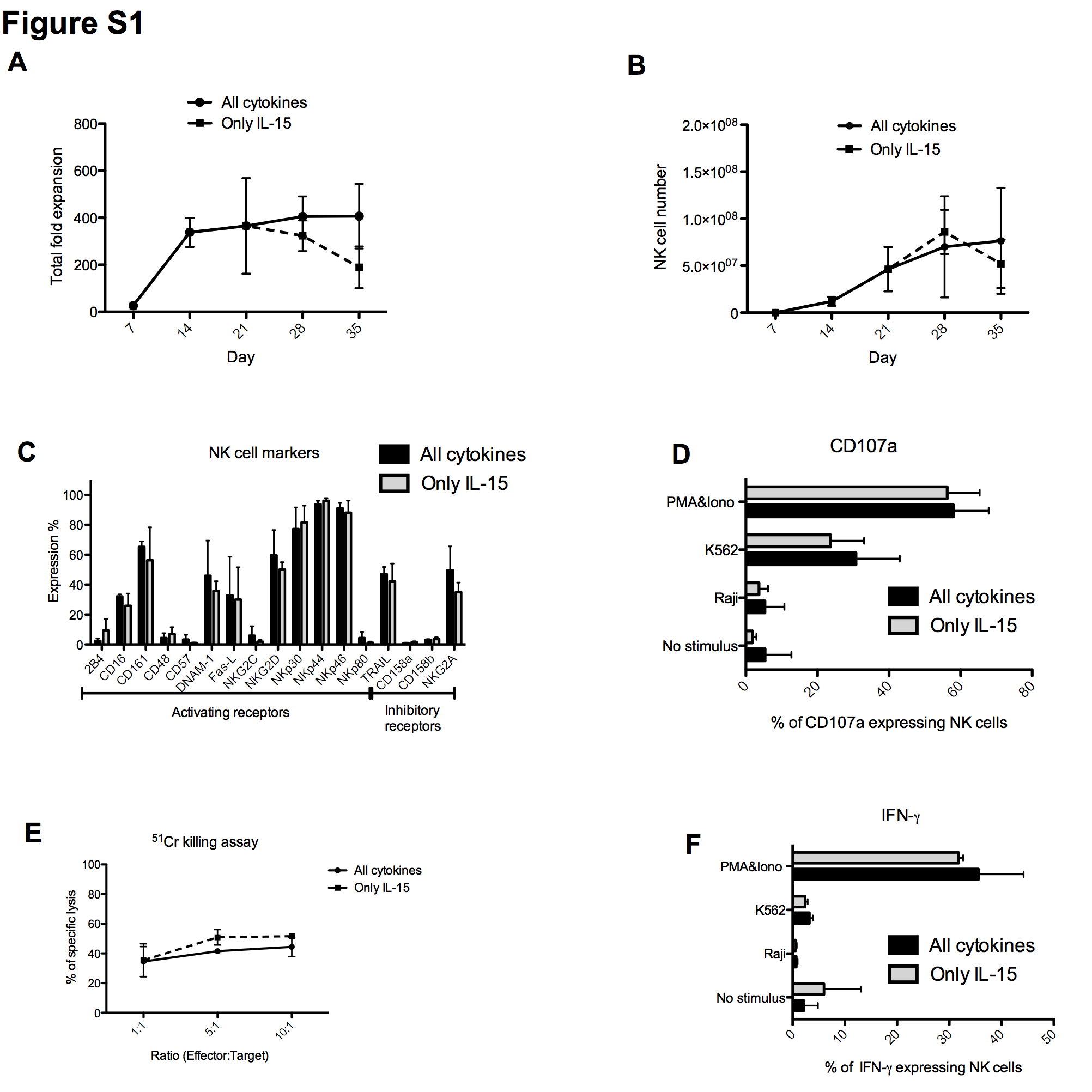

Supplement: Figure S1 — NK cell production from fresh CBCD34+ cultures using different cytokine cocktails. (A) Total fold expansion and (B) cell number of CD3−CD56+ cells of fresh CBCD34+ cultures using all cytokines (n = 3, solid line) or only IL-15 (n = 3, dotted line). (C) Expression of NK cell markers by NK cells from fresh CBCD34+ cultures using all cytokines (n = 3) or only IL-15 (n = 3). (D) Degranulation assay using CD107a on NK cells from fresh CBCD34+ cultures using all cytokines (n = 3) or only IL-15 (n = 3). (E) NK cells from fresh CBCD34+ cultures using all cytokines (n = 3) or only IL-15 (n = 3) were co-incubated with 51Cr-labeled K562 cells at different effector-target ratios in a standard 4 h 51Cr-release assay. (F) Produciton of IFN-γ by NK cells from fresh CBCD34+ cultures using all cytokines (n = 3) or only IL-15 (n = 3). (TIFF) [file pone.0087086.s001.tif]

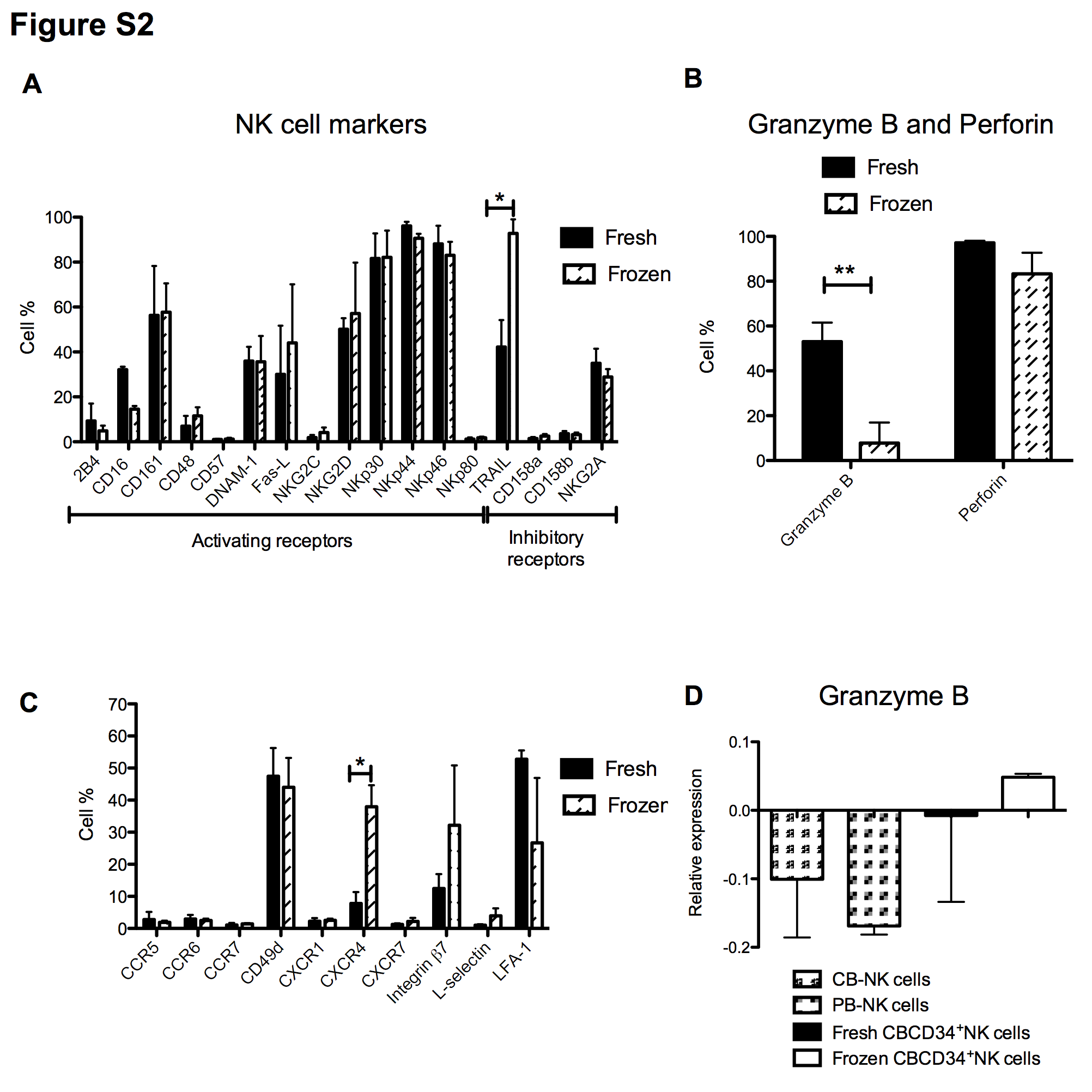

Supplement: Figure S2 — Characterization of fresh and frozen CBCD34+-NK cells. The graph shows expression of (A) NK cell markers, (B) intracellular granzyme B and perforin and (C) chemokine receptors by NK cells from fresh (n = 3) and frozen (n = 4) CBCD34+ cultures. (D) Transcriptional analysis of granzyme B mRNA in NK cells from different sources. Values were normalized using three reference genes. Higher ratio values correspond to less mRNA expression. Mann-Whitney test was performed. * P<0.05, ** P<0.005. (TIFF) [file pone.0087086.s002.tif]

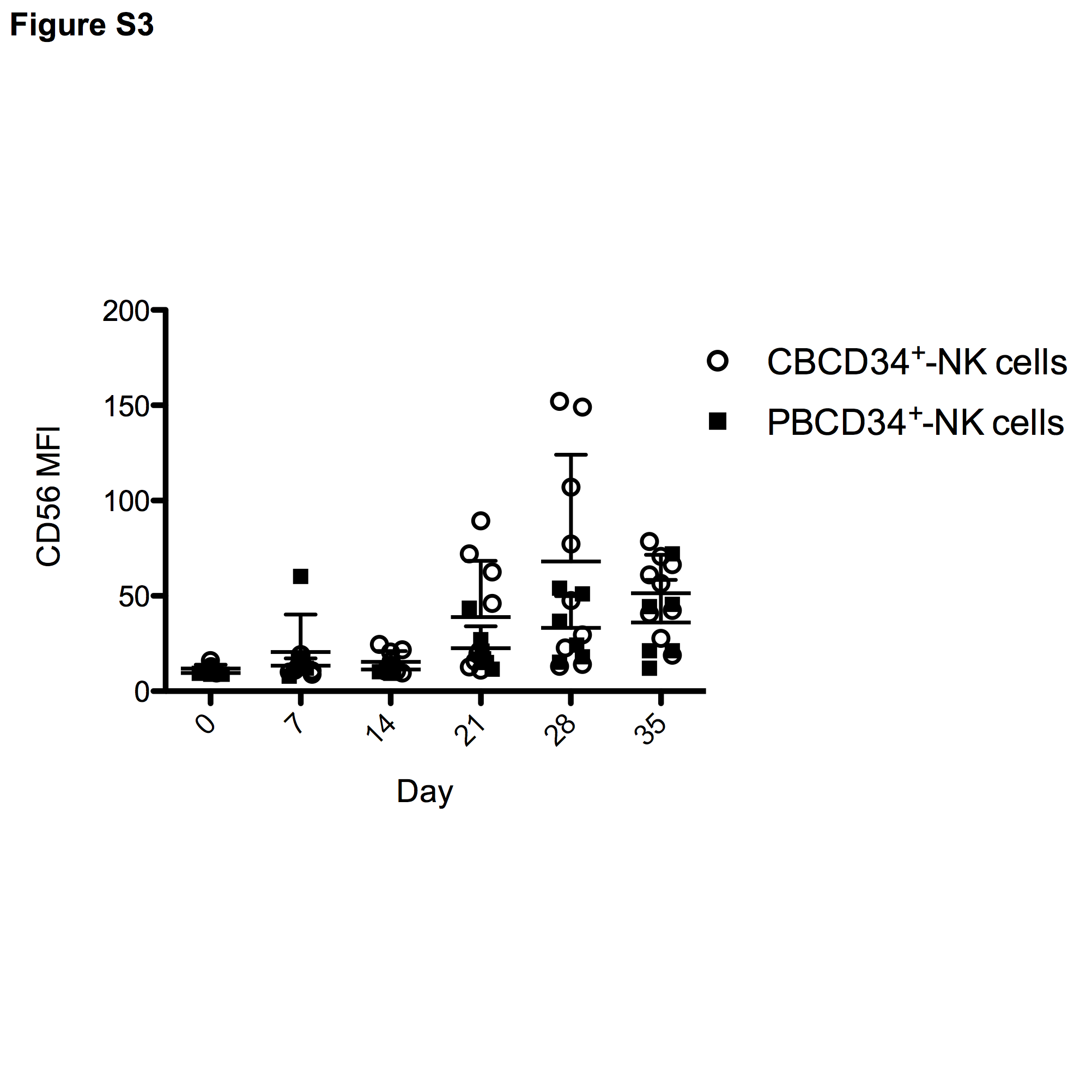

Supplement: Figure S3 — Expression of CD56 during HSC cultures. Expression of CD56 as measured by MFI by NK cells from CBCD34+ (n = 9) and PBCD34+ (n = 6) cultures at different time points. (TIFF) [file pone.0087086.s003.tif]

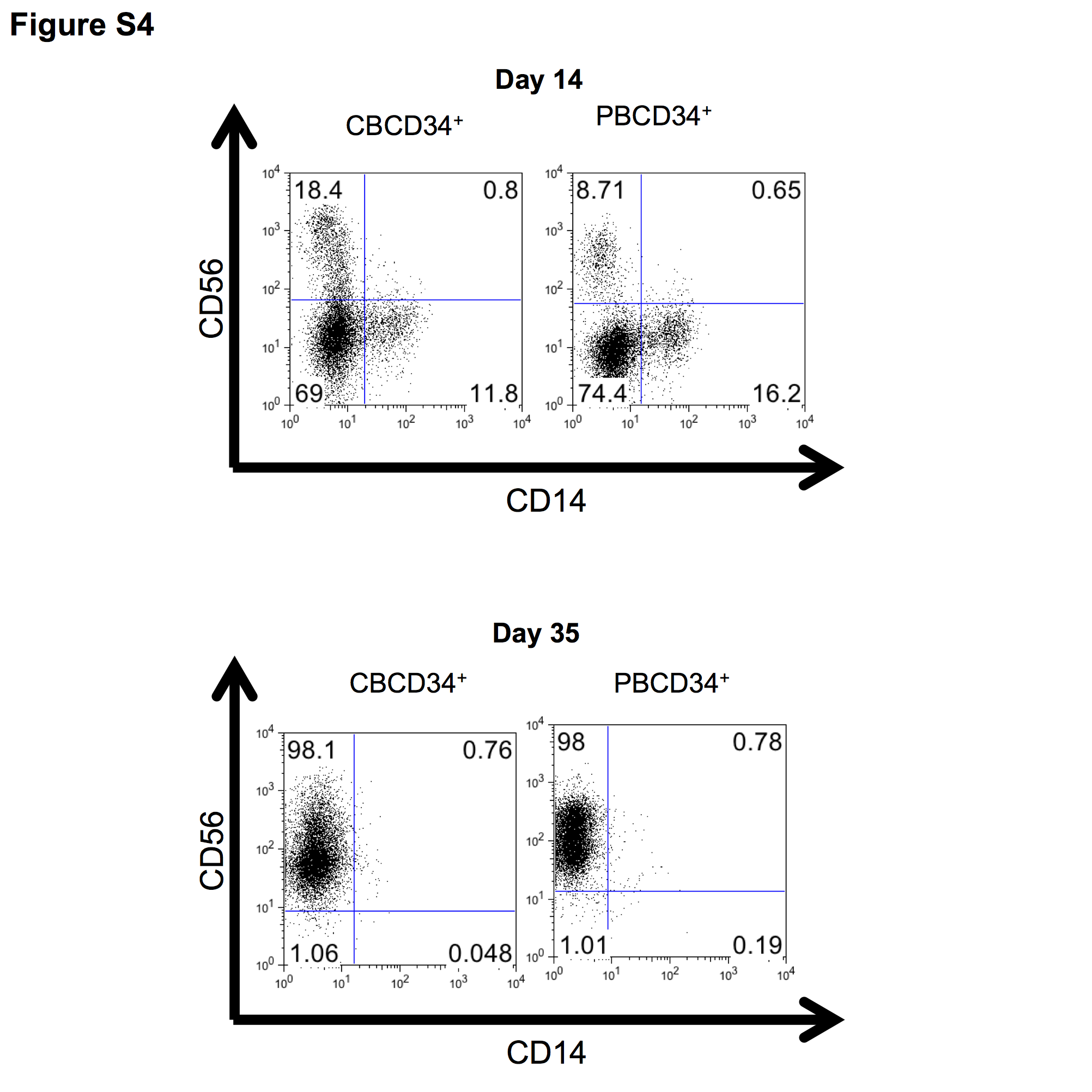

Supplement: Figure S4 — Expression of CD14 during HSC cultures. A representative FACS plot (CD56 vs CD14) from CBCD34+ and PBCD34+ cultures at days 14 and 35 showing expression of the monocyte marker CD14. (TIFF) [file pone.0087086.s004.tif]

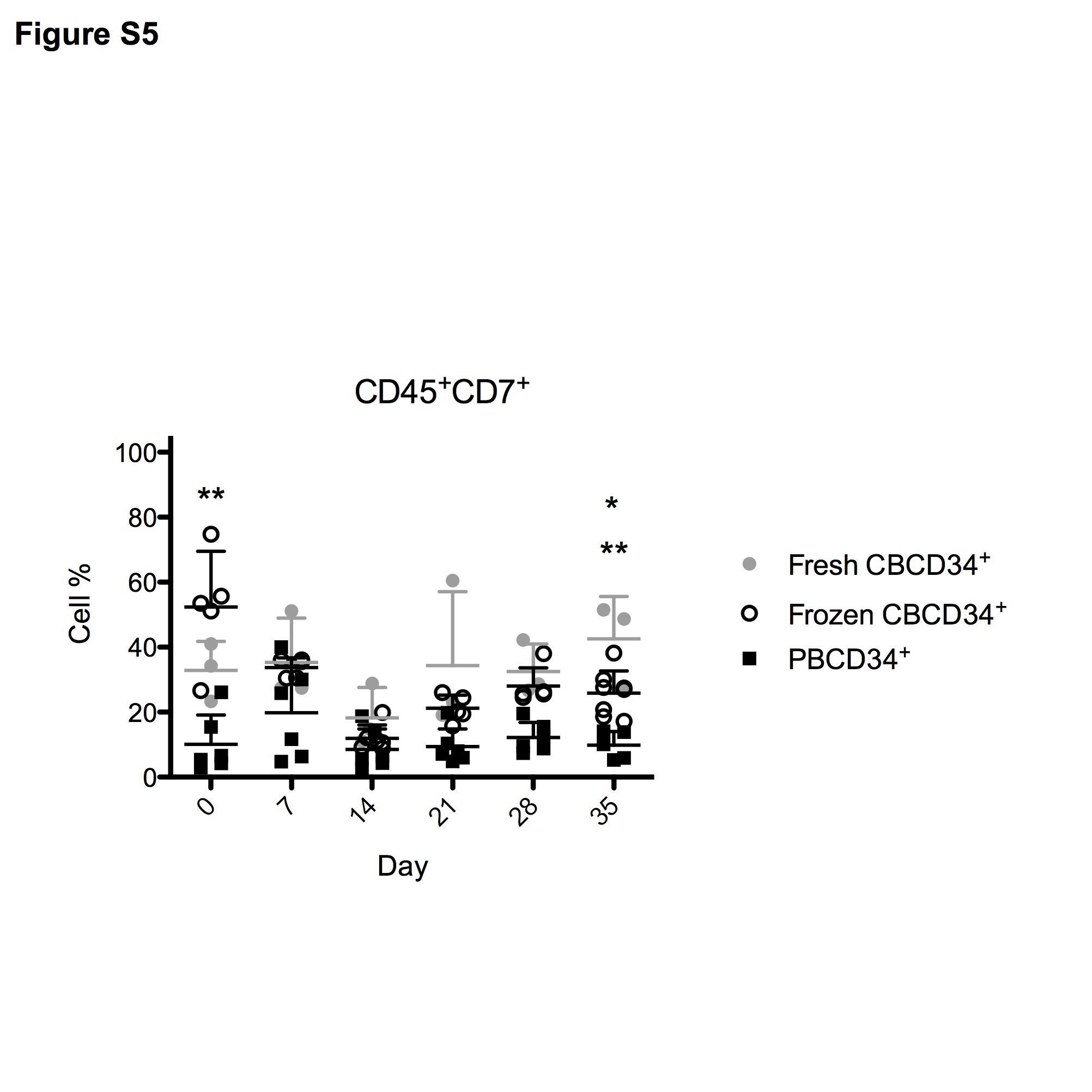

Supplement: Figure S5 — Frequency of CD45+CD7+ cells during HSC cultures. Percentages of CD45+CD7+ progenitor cells in fresh (n = 3) and frozen CBCD34+ (n = 9) and PBCD34+ (n = 6) cultures at different time points. (TIFF) [file pone.0087086.s005.tif]

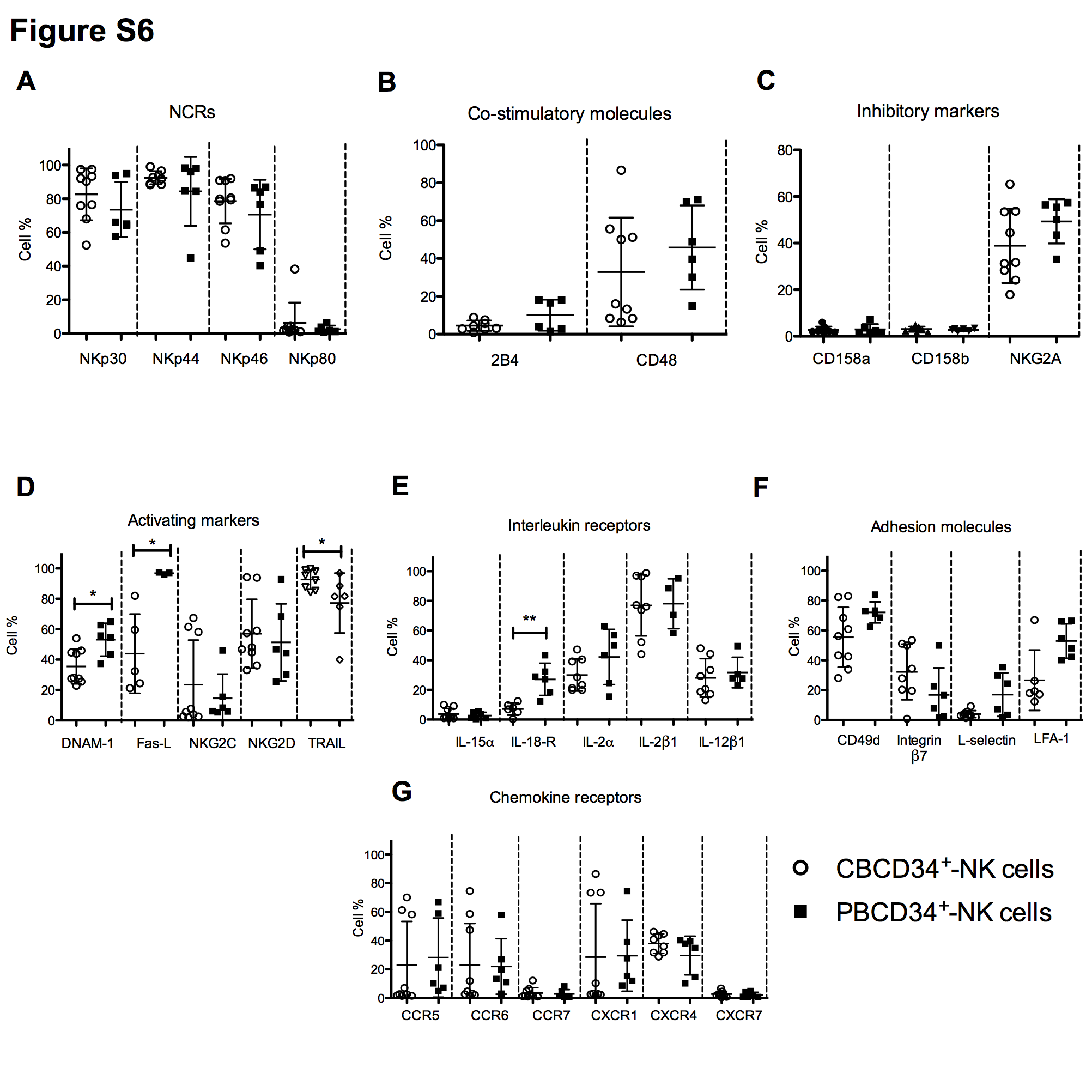

Supplement: Figure S6 — Phenotypic characterization of NK cells from CBCD34+ and PBCD34+ cultures. NK cells from CBCD34+ (n = 9, open circles) and PBCD34+ (n = 6, black squares) cultures were harvested at day 35 and stained with antibodies against the indicated surface antigens. For each marker, the median and standard deviation is presented for (A) Natural cytotoxicity receptors (NCRs), (B) co-stimulatory molecules, (C) inhibitory markers, (D) activating markers, (E) interleukin receptors, (F) adhesion molecules and (G) chemokine receptors on CD56+CD3− cells from both cultures. The statistical analysis was performed using Mann-Whitney test. * P<0.05, ** P<0.005. (TIFF) [file pone.0087086.s006.tif]

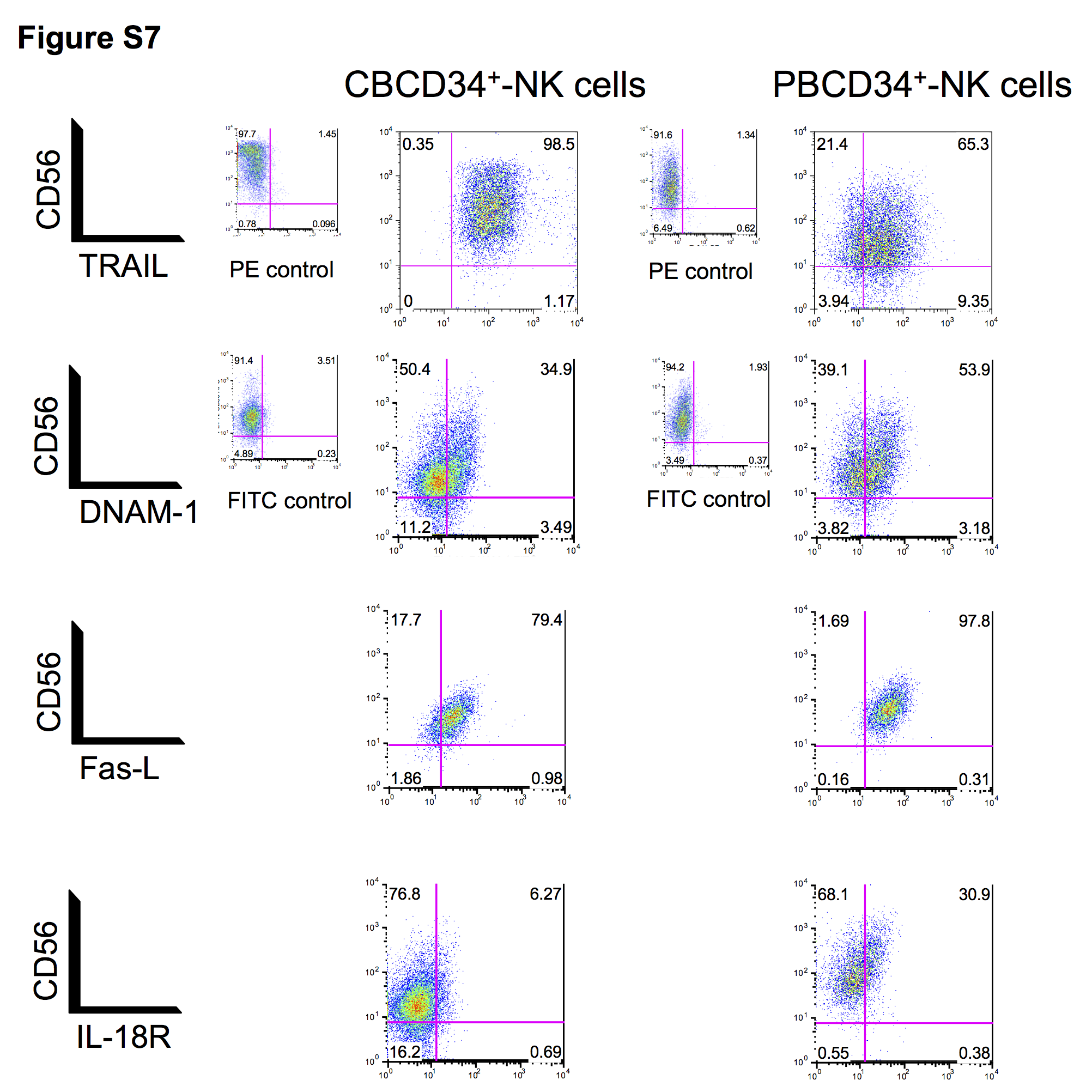

Supplement: Figure S7 — Expression of TRAIL, DNAM-1, Fas-L and IL-18R by NK cells from CBCD34+ and PBCD34+ cultures. Representative FACS plots of CD56 vs CD14, CD56 vs DNAM-1, CD56 vs Fas-L and CD56 vs IL-18R of NK cells from CBCD34+ and PBCD34+ cultures. (TIFF) [file pone.0087086.s007.tif]

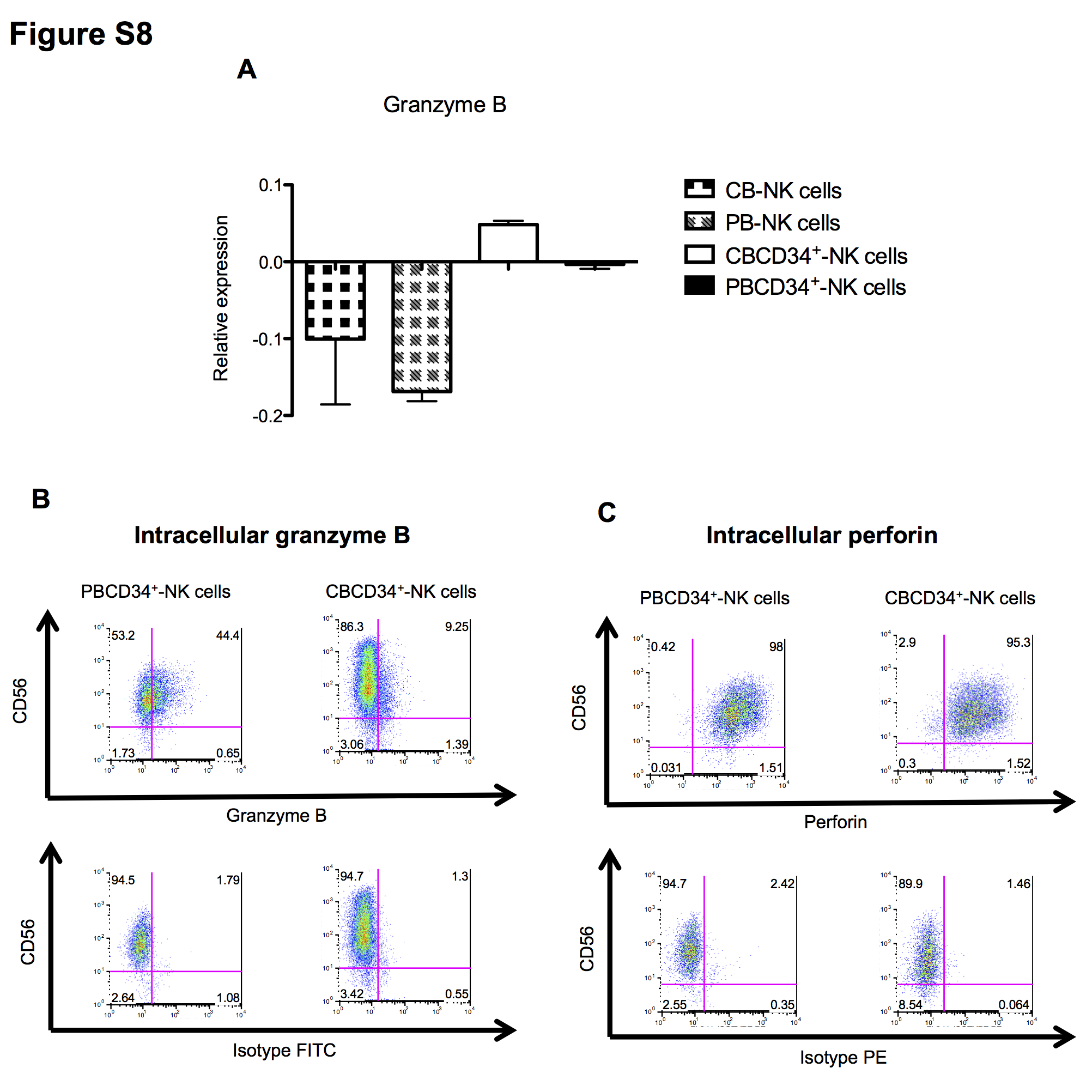

Supplement: Figure S8 — Granzyme B expression by NK cells from CBCD34+ and PBCD34+ cultures. (A) Transcriptional analysis of granzyme B mRNA in NK cells from CB, PB, CBCD34+ cultures and PBCD34+ cultures. Values were normalized using three reference genes. Higher ratio values correspond to less mRNA expression. Representative FACS plots of CD56 vs Granzyme B (B), CD56 vs Perforin (C) or the corresponding isotype control of NK cells from CBCD34+ and PBCD34+ cultures. (TIFF) [file pone.0087086.s008.tif]

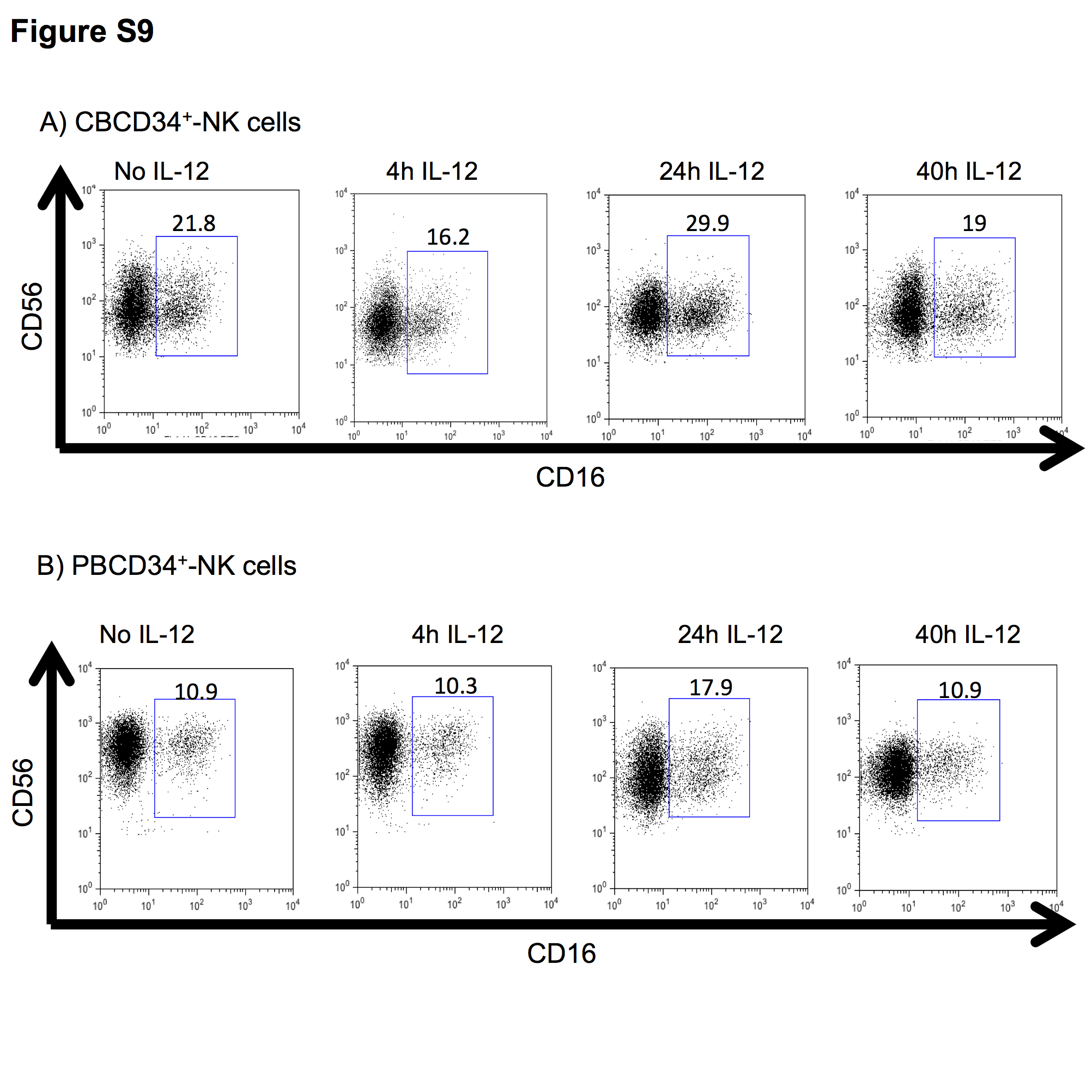

Supplement: Figure S9 — Effect of IL-12 on CD16 expression by the differentiated NK cells. The figure shows a representative example of CD56+CD3− cells from (A) CBCD34+ and (B) PBCD34+ cultures prior to and after incubation with IL-12 for 4, 24 or 40 h. The plots show CD56 vs CD16 for each time point. Percentages shown represent CD56+CD16+ cells. (TIFF) [file pone.0087086.s009.tif]

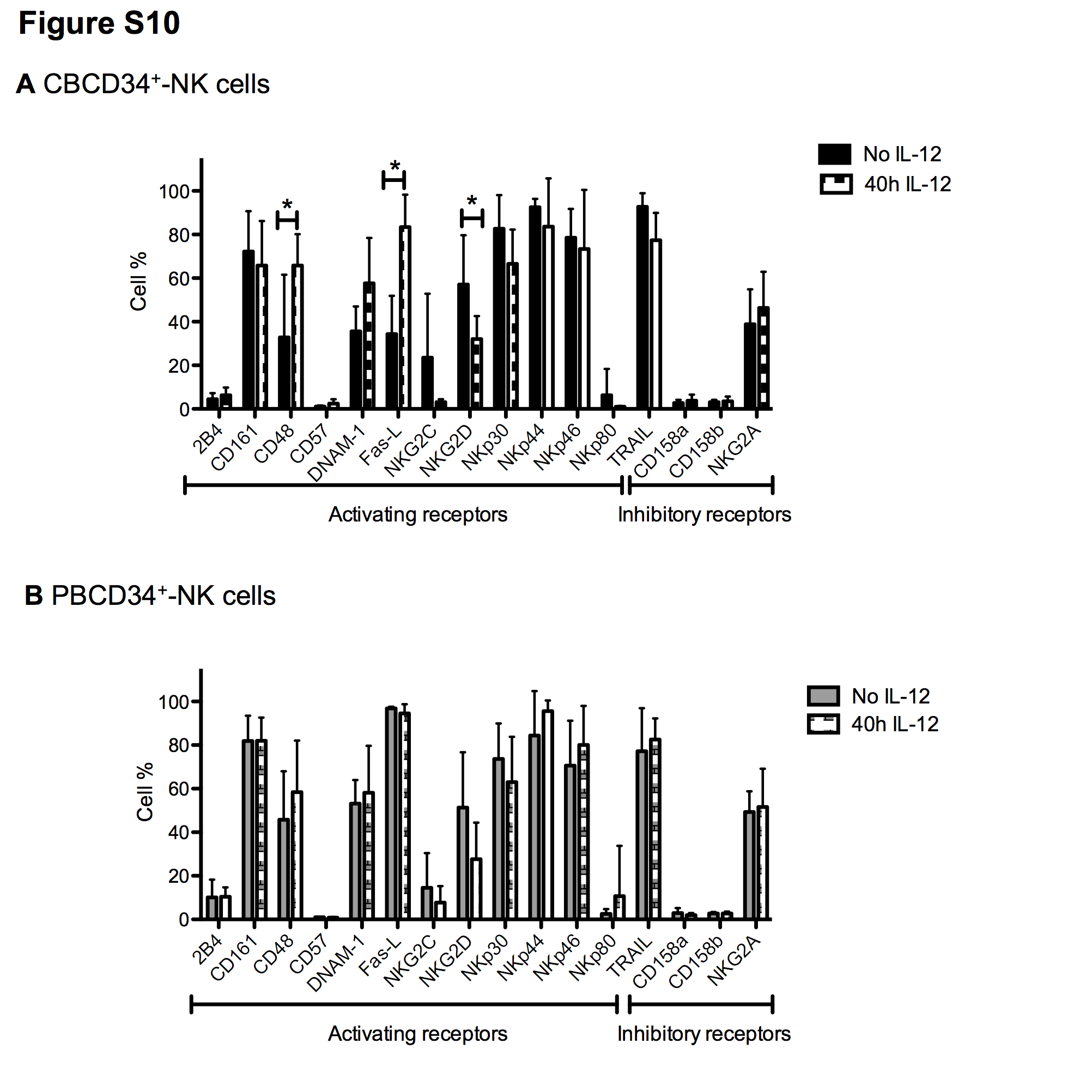

Supplement: Figure S10 — Effect of IL-12 on the expression of activating and inhibitory receptors by differentiated NK cells. NK cells from (A) CBCD34+ (n = 9) and (B) PBCD34+ (n = 6) cultures were incubated with IL-12 for 40 h. After incubation, cells were collected and labelled with antibodies against the indicated surface antigens. Statistical analysis was performed using Mann-Whitney test. * P<0.05. (TIFF) [file pone.0087086.s010.tif]

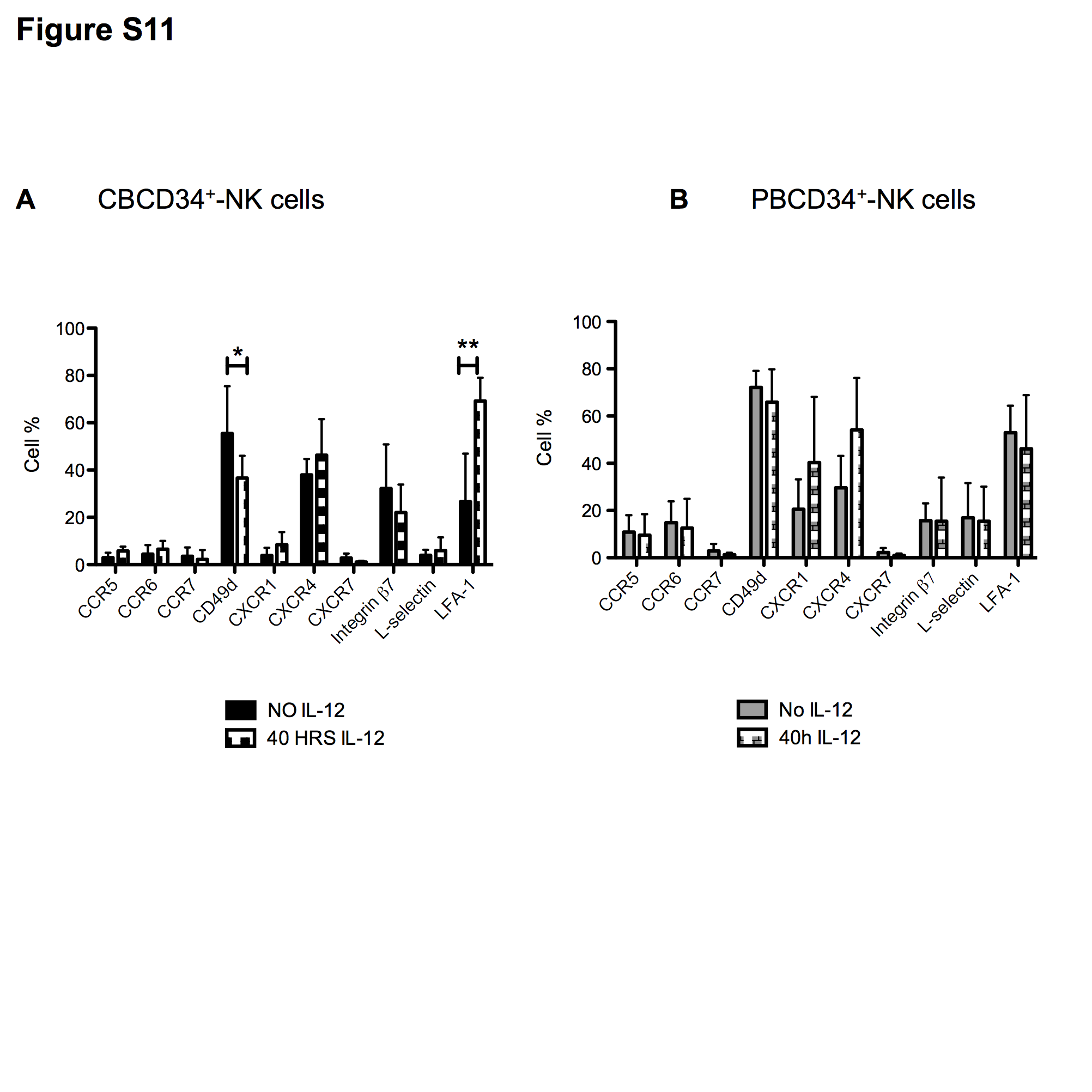

Supplement: Figure S11 — Effect of IL-12 on the expression of chemokine receptors and adhesion molecules by the differentiated NK cells. NK cells from (A) CBCD34+ (n = 9) and (B) PBCD34+ (n = 6) cultures were incubated with IL-12 for 40 h. After incubation, cells were collected and labelled with antibodies against the indicated surface antigens or an isotype control. Statistical analysis was performed using Mann-Whitney test. * P<0.05. (TIFF) [file pone.0087086.s011.tif]
